# Nutritional Function and Flavor Evaluation of a New Soybean Beverage Based on *Naematelia aurantialba* Fermentation

**DOI:** 10.3390/foods11030272

**Published:** 2022-01-20

**Authors:** Tao Sun, Hao Jiang, Kai Yang, Xingkai Li, Shiyu Wang, Haoyu Yao, Rui Wang, Sha Li, Yian Gu, Peng Lei, Hong Xu, Dafeng Sun

**Affiliations:** 1 State Key Laboratory of Materials-Oriented Chemical Engineering, College of Food Science and Light Industry, Nanjing Tech University, Nanjing 211816, China; sun-tao@njtech.edu.cn (T.S.); jianghao@njtech.edu.cn (H.J.); 201961119005@njtech.edu.cn (K.Y.); 201921019042@njtech.edu.cn (X.L.); 201921019081@njtech.edu.cn (S.W.); 201921019097@njtech.edu.cn (H.Y.); ruiwang2013@njtech.edu.cn (R.W.); lisha@njtech.edu.cn (S.L.); yian.gu@hotmail.com (Y.G.); 2 Kunming Edible Fungi Institute of All China Federation of Supply and Marketing Cooperatives, Kunming 650032, China; sdafeng@163.com

**Keywords:** sensory evaluation, hierarchical cluster analyses, volatile organic compounds, GC-MS, GC-IMS, principal component

## Abstract

The soy beverage is a healthy product rich in plant protein; however, its unpleasant flavor affects consumer acceptance. The aim of this study was to determine the feasibility of using *Naematelia aurantialba* as a strain for the preparation of fermented soybean beverages (FSB). Increases in Zeta potential, particle size, and viscosity make soy beverages more stable. We found that nutrient composition was increased by fermenting *N. aurantialba*, and the antioxidant activity of soybean beverages significantly increased after 5 days of fermentation. By reducing the content of beany substances such as hexanal and increasing the content of 1-octen-3-ol, the aroma of soybean beverages fermented by *N. aurantialba* changed from “beany, green, and fatty” to “mushroom and aromatic”. The resulting FSB had reduced bitterness but considerably increased sourness while maintaining the fresh and sweet taste of unfermented soybean beverages (UFSB). This study not only provides a theoretical basis for the market promotion of FSB but also provides a reference for basidiomycetes-fermented beverages.

## 1. Introduction

With the recent enhancement of nutritional, healthy, and green dietary ideas, plant protein drinks are gaining increasing attention, and their development remains of ongoing interest in the beverage industry [1]. Soybeans are one of the most important sources of plant proteins due to their high protein and essential amino acid content [2]. The soy beverages are considered alternatives to milk, especially for individuals allergic to milk proteins, intolerant of lactose, or on a vegetarian diet [2]. Commercialized beverages should not only be rich in nutrients but also have a good flavor [3]. However, the beany flavor of soy beverages is the main factor limiting their acceptance by consumers [1,2,4]. The compounds causing this beany flavor are mainly decomposition products of soybean lipids catalyzed by lipoxygenases (LOX) and hydroperoxide lyases [5]. Generally, two methods are used to reduce the beany flavor of soy beverages [6]. First, LOX knockout soybean cultivars are used [7]. However, previous research has revealed that removing complete LOX isozymes from soybeans can have a negative impact on the plant’s defense mechanisms [8]. Second, methods such as temperature control, acid treatment, enzyme treatment, and supercritical carbon dioxide extraction have been employed to remove flavors from processing conditions, but the high energy consumption and cost of such methods make the preparation cost of soybean beverages too high to benefit commercialization [9].

Fermentation using probiotics or edible fungi is considered an inexpensive and safe method of improving the nutritional composition and flavor compounds of plant-based foods and increasing their shelf stability [10,11,12,13]. At present, Lactobacillus species, such as *L. plantarum*, *L. bulgaricus*, and *L. paracasei*, are used to improve the sensory properties of soymilk and reduce or mask the beany flavor of soybeans [14]. However, the development of plant-based soybean beverage varieties, particularly fermentation type microbial species diversity, will be hindered if only strains of the lactic acid bacteria genus are used as fermented soybean beverages. As a result, researchers all around the world are seeking strategies to overcome this constraint by employing alternative probiotics. The products being developed involve the use of various types of bacteria and fungi, particularly edible fungi, in the same medicines and foods. Previous studies have found that fermenting soybean beverages with basidiomycetes has great application potential. *Phellinus igniarius* and *Agrocybe cylindracea* not only showed good growth conditions in soy beverages, but also improved antioxidant properties and inhibited epidermal tumor proliferation in mice [15]. *Ganoderma lucidum* was used to ferment soy beverages to improve their health properties, and *Lycoperdon pyriforme* was used to adjust their aroma and eliminate the beany flavor [11,12,13,14,15,16]. However, there are still some problems facing basidiomycete-fermented soybean beverages; specifically, most of the currently used strains are mycelia, which degenerate easily and result in poor quality of the final fermented products.

*Naematelia aurantialba*, also known as *Tremella aurantialba*, is an indigenous Chinese edible and medicinal fungus [17]. Previous research has found that polysaccharides, saponin, phenolic, and flavonoid compounds in *N. aurantialba* are responsible for antioxidation, antiinflammatory, antitumor, and immunomodulatory effects [18,19,20,21]. In the absence of an associate, *N. aurantialba* acts as a basidiomycete whose spores multiply in an outgrowth manner and do not develop into mycelium [22,23]. Our previous study showed that the most favored nitrogen source for exopolysaccharide production by *N. aurantialba* NX-20 was soybean protein, which means that it could not only utilize soybean beverage for growth but also secrete its most active substance, *N. aurantialba* polysaccharide (NAPS) [24,25]. While ultra-high molecular weight NAPS perform multiple biological activities, they also act as a water-soluble pseudoplastic fluid to increase the stability and viscosity of soybean beverages. Thus, soybean beverages fermented with NAPS do not require the incorporation of complex and expensive stabilizers, resulting in reduced production costs. In addition, the flavor of fermented liquor from *N. aurantialba* is pleasant and has a high potential for application in the removal or masking of the beany flavor of fermented soybean beverages [26].

Therefore, in this study, an unfermented soybean beverage (UFSB) was fermented by *N. aurantialba* to produce a fermented soybean beverage (FSB). The physicochemical properties and nutritional components of the soybean beverages were examined, and their antioxidant capacity was investigated. In addition, HS-GC-IMS and HS-SPME-GC-MS were utilized to explore the changes in volatile odor in soybean beverages. Finally, the aroma and taste characteristics of the soybean beverage were evaluated by sensory evaluation and electronic tongue technology.

## 2. Materials and Methods

### 2.1. Preparation of Soy Beverages

To prepare the UFSB from fresh soybeans, the soybeans were washed with distilled water and soaked for 12 h at 25 °C. The beverage was then prepared using a soybean milk machine at a solid–liquid ratio of 1:5 (mass ratio). It was then filtered through four layers of cotton gauze and autoclaved at 105 °C for 20 min.

To prepare the fermented soy beverage (FSB) from fresh soybeans, the isolated strain, *N. aurantialba* NX-20, which had been preserved in the China General Microbiological Culture Collection Center (CGMCC 18588), was inoculated into the seed medium (potato dextrose broth) and cultured at 25 °C for 3 d. The seed liquid was then transferred to 100 mL of fermentation medium at 10% inoculation and cultured at 25 °C for 5 d. The fermentation medium consisted of 4 g of glucose and 100 mL of UFSB. After fermentation, the broth was boiled for 30 min to inactivate the *N. aurantialba* spore cells, and then the samples were frozen at −80 °C until ready for further analysis. In addition, the spread plate method was used to detect the quality of microorganisms in unsterilized soy fermented beverages. The phosphate-buffered saline (PBS) was used to make gradient dilutions of the fermentation broth. The number of cells was then counted after potato dextrose agar (PDA) culture (4 d, 5 °C). The amount of *N. aurantialba* in unsterilized soy fermented beverages was approximately 10^9^ ± 5 × 10^7^ CFU/mL.

### 2.2. Changes in Physicochemical Properties of Soy Beverages

The particle size and zeta potential of the samples were analyzed using a nanoparticle potentiometer (NICOMP Z3000, Port Richey, FL, USA) [27]. The test sample was diluted with de-ionized water (1:12.5) and stirred at 300 rpm for approximately 10 min before analysis. The average size of the samples was determined based on polarization intensity difference scattering [27].

The viscosity of the soybean beverage was measured using an Ubbelohde viscometer (NDJ-8S, Nanjing, China) in a 25 °C water bath [28].

A HunterLab UltraScan PRO (Hunter Associate Laboratory Inc., Reston, VA, USA) colorimeter was used to examine the samples [29]. The samples were placed in optical glass cells with a fixed route length of 50 mm and a 25 mm aperture for reflectance measurement (Large Area View—LAV). The average of five readings was used to arrive at the findings. The CIELab L* (lightness), a* (redness), and b* (yellowness) values are used by the UltraScan PRO to measure the reflected color of food products.

A digital pH meter (PHS-3, Shanghai INESA Scientific Instrument Co., Ltd., Shanghai, China) was used to measure the pH.

### 2.3. Analysis of Nutrients

The total soluble protein in a sample was measured using the Bradford method according to the manufacturer’s instructions [30].

The total fat in a sample was measured using a Soxhlet apparatus, which is based on the Soxhlet extractor method [11].

The total sugar content in the sample was determined using the phenol sulfuric acid method. The reducing sugars were determined using the DNS method. Determination of glucose concentration was conducted using the SBA-40c biosensor (Biological Institute of Shandong Academy of Science, Jinan, China) [24].

The amount of total dietary fiber in the sample was determined using the enzymatic gravimetric method [30].

The total acid content in a sample acid-base titration was determined based on the enzymatic gravimetric method [30].

Free amino acids in the samples were analyzed using an automated amino acid analyzer (S-433D, Sykam, Munich, Germany) [31].

The Folin–Ciocalteu method and aluminum chloride were used to determine the total flavonoid and phenolic contents, respectively; each method was performed according to the manufacturer’s instructions on the kits (Nanjing JianCheng Bioengineering Institute, Nanjing, China) [32].

The NAPS content in the samples was determined by HPLC-GPC. Based on the detection of NAPS by HPLC (Shimadzu 20A, Tokyo, Japan), two OHpak SB-806M HQ columns (8.0 mm × 300 mm, Shodex, Tokyo, Japan) were used in succession [24]. The mobile phase consisted of ultrapure water at a flow rate of 1.0 mL/min. Detection was performed using an evaporative light scattering detector (ELSD, ELSD-16) with a drift tube temperature of 60 °C and ventilation rate of 3.0 L/min. The NAPS content was calculated according to the standard linear equation formed by different concentrations of NAPS standards [24].

### 2.4. Analysis of Antioxidant Capacity

ABTS (2, 2′-azino-bis (3-ethylbenzothiazoline-6-sulfonic acid), DPPH (2,2-Diphenyl-1-picrylhydrazyl), Fenton, and pyrogallol autoxidation methods were used to analyze ABTS radical scavenging activity, DPPH radical scavenging activity, scavenging rate of hydroxyl radical activity, and scavenging rate of superoxide anion radical activity. Each method was performed according to the manufacturer’s instructions on the kits (Nanjing JianCheng Bioengineering Institute, Nanjing, China) [33,34].

### 2.5. Electronic Tongue Measurement

The flavor profiles of the soy beverages were analyzed using a potential electrotongue (SA402B; Insent, Tokyo, Japan) [35,36]. The sensor of the e-tongue was first pre-equilibrated using 0.01 moL·L^−1^ of sodium chloride, 0.01 moL·L^−1^ of sodium glutamate, and 0.01 moL·L^−1^ hydrochloric acid, prior to detection. The e-tongue had an acquisition time of 120 s for each part of the sample, one data collection per second, nine acquisition times for each part of the sample, then 30 s of washing, and the results were given as the average of three data points.

### 2.6. Analysis of Volatile Compounds

#### 2.6.1. Headspace Gas Chromatography-Ion Mobility Spectrometry (HS-GC-IMS) Analysis

The volatile organic compounds (VOCs) of the UFSB and FSB were analyzed by HS-GC-IMS analysis based on FlavourSpec^®^ Flavor analyzers (Gesellschaft für Analytische Sensorsysteme mbH, Dortmund, Germany) and an Agilent 490 gas chromatograph (Agilent Technologies, Palo Alto, CA, USA) [37,38]. Approximately 1 g of sample was weighed and placed in a 20 mL headspace bottle and analyzed by an autosampler after incubation at 60 °C for 20 min (injection volume—500 μL, injection needle temperature—85 °C). The GC analytical column was a FS-SE-54-CB-1 capillary column (15 m × 0.53 mm, RESTEK, Bellefonte, PA, USA). The carrier gas, which was nitrogen, followed a programmed flow rate: initially the flow rate was 2 mL/min for 2 min, and then it was increased to 10 mL/min for 8 min, 100 mL/min for 10 min, and finally 150 mL/min for 10 min. The total GC run time was 30 min. The samples were separated in a column at 60 °C and then ionized in an IMS ionization chamber using a 3H ionization source (300 MBq activity). A 9.8 cm drift tube with a constant voltage (5 kV at 45 °C) and a nitrogen flow of 150 mL/min was used. Each spectrum was reported as an average of 12 scans. Qualitative and quantitative analyses of VOCs were performed using the National Institute of Standards and Technology (NIST) database built into the software, the IMS database, and reference materials [38].

#### 2.6.2. Headspace Solid-Phase Microextraction Gas Chromatography–Mass Spectrometry (HS-SPME-GC-MS) Analysis

The volatile compounds in the FSB and UFSB samples were determined by HS-SPME-GC-MS [39]. One gram of the sample was transferred to a 20 mL headspace bottle (Agilent, Palo Alto, CA, USA) containing a saturated solution of NaCl to inhibit any enzyme reactions. The vials were sealed using crimp-top caps with a TFE-silicone headspace septa (Agilent). During the SPME analysis, each vial was placed at 60 °C for 10 min, and then a 65 µm divinylbenzene/carboxen/polydimethylsiloxane fiber (Supelco, Bellefonte, PA, USA) was exposed to the headspace of the sample for 20 min at 60 °C. After sampling, desorption of the VOCs from the fiber coating was carried out in the injection port of the GC apparatus (Model 7890 B; Agilent) at 250 °C for 5 min in splitless mode. The identification and quantification of VOCs was carried out using an Agilent Model 7890B GC and a 7000D mass spectrometer (Agilent), equipped with a 30 m × 0.25 mm × 1.0 μm DB-5MS (5% phenyl-polymethylsiloxane) capillary column. Helium was used as the carrier gas at a linear velocity of 1.0 mL/min. The temperatures of the injector and detector were kept at 250 and 280 °C, respectively. The oven temperature was set to 40 °C (5 min) and increased at 6 °C/min to 280 °C, and maintained for 5 min. Mass spectra were recorded in the electron impact ionization mode at 70 eV. The temperatures of the quadrupole mass detector, ion source, and transfer line were set to 150, 230, and 280 °C, respectively. Mass measurements were taken at 1 s intervals. Spectra were scanned in the range of *m*/*z* 30–350 amu [40].

The identification of volatile compounds was achieved by comparing the mass spectra with the data system library and linear retention index. The flavor description of volatile compounds was determined using the volatile compounds in food (VCF) database (https://www.vcf-online.nl/VcfHome.cfm, accessed on 10 July 2021), a good subject company information system (http://www.thegoodscentscompany.com/index.html, accessed on 10 July 2021), and references [11,41].

### 2.7. Analysis of Relative Odor Activity Value

The relative odor activity value (*ROAV*) was calculated using the following equation

(1) $$ROAV\approx 100\times \frac{C_{sample}}{C_{standard}}\times \frac{T_{standard}}{T_{sample}}$$

where *C_sample_* and *T_sample_* represented each volatile component’s relative content and odor threshold, respectively. *C_standard_* and *T_standard_* represented the relative contents and odor thresholds, respectively, of the constituents that contributed most to the total odor in the sample [41].

### 2.8. Sensory Evaluation

The odors of the unfermented and fermented samples, including sour, mushroom, aromatic, fatty, green, and beany flavors, were evaluated by 20 experienced, trained assessors (10 males and 10 females). The intensity of each category was recorded based on a 7-point system, with 1 being low intensity and 7 being high intensity [41].

### 2.9. Statistical Analysis

All the experiments were performed using at least three independent samples. The SPSS software (version 19.0; SPSS Inc., Chicago, IL, USA) was used for statistical analysis and the results are expressed as the mean ± SD. The metabolites were characterized by analyzing mass spectra using the software Qualitative Analysis Workflow B.08.00. One-way analysis of variance and the least significant difference test were used, and the significance level is 0.05 (α = 0.05).

In addition, for GC-IMS analysis, the laboratory analytical viewer was used to visualize the analyzed spectra, where each point referred to a VOCs that could be quantitatively analyzed by establishing a standard curve and reporter plugin for direct comparison of spectral differences between samples (two-dimensional top view and three-dimensional spectra). The gallery plot plugin was used to perform the comparison of fingerprinting patterns between samples.

Principal component (PCA) and hierarchical cluster analyses were performed using the statistical function prcomp within the R package (version 4.2, www.r-project.org, accessed on 1 July 2021) [42].

The sensory analysis was carried out on three parallel samples for each variable, and the data was expressed as mean value ± standard error. 

## 3. Results and Discussion

### 3.1. Physicochemical Properties and Nutrient Composition of the Soybean Beverage

#### 3.1.1. Analysis of Physicochemical Properties of the Soybean Beverage

The physical and chemical properties of UFSB and FSB are shown in Table 1. The mean particle sizes of UFSB and FSB were 198 nm and 926 nm, respectively. The increased particle sizes of FSB might be because fermentation produced NAPS with molecular weights of up to 2924.6 kDa, or because some proteins produced by fermentation were denatured during sterilization [43]. Furthermore, the Zeta potentials of UFSB and FSB were −12.41 and −32.54 mv, respectively. The term “Zeta potential” is widely used to indicate the degree of electrostatic repulsion between adjacent, similarly charged, colloidal particles in suspension, with higher surface charges enhancing electrostatic repulsion between droplets and overcoming van der Waals’ forces and hydrophobic attractions [44]. The absolute Zeta potential value increased in FSB, indicating that after *N. aurantialba* fermentation, the stability of the soy beverage increased [44]. Pectin, chitosan, carboxymethyl cellulose, and other polysaccharides are commonly employed as stabilizers and thickeners to alter the zeta potential, average particle size, and viscosity of drinks to improve their stability, and NAPS may have a similar function [45,46]. The NAPS is an anionic polysaccharide and the polysaccharide protein complex generated by it binds with proteins, increasing the electrostatic and steric repulsion forces between the soybean beverage droplets and, thereby, increasing the viscosity [24]. To test the latter hypothesis, we compared the viscosities of both soybean beverages and found that the viscosities of FSB and UFSB were 804 mPa·s and 3.4 mPa·s, respectively. This indicated that NAPS indeed increased the viscosity of the soybean beverages following fermentation. Such an increase in viscosity might improve beverage stability but may be distasteful to consumers [47]. Taking this into consideration, FSB in the market can be diluted using purified water. The color values and appearances of both beverages are shown in Table 1. The a*-value (red/green) and b*-value (blue/yellow) of the color measurement were statistically significant, but not the lightness of L*. This meant that the UFSB had gotten redder and bluer than the FSB. However, the hue shift was hardly noticeable to the naked eye, and all beverages appeared “milk white, faint yellow.” The pH of FSB and UFSB was 4 and 6, respectively. This indicates that the soybean beverage fermented with *N. aurantialba* generated more acidic chemicals, resulting in an acidic taste due to increased glucuronic acid synthesis. Lactic acid bacteria-fermented milk with a pH of around 4 is available on the market [48].

#### 3.1.2. Analysis of Soy Beverage Nutrient Composition

The levels of protein, fatty acid, total sugar, reducing sugar, dietary fiber, total acid, moisture, ash content, total amino acids, total polyphenols, total flavonoids, and NAPS in the FSB and UFSB are shown in Table 1. The protein content of FSB (17.0 ± 1.3 g/kg) was lower than that of UFSB (28.0 ± 1.5 g/kg), respectively. It may be that the proteins in the soybean beverage were used by *N. aurantialba* as a nitrogen source for the fungi’s growth, which might explain the higher levels of total free amino acids in FSB than in UFSB. No significant differences in the levels of fatty acids or dietary fiber were observed before or after fermentation, which is consistent with previous studies using *L. pyriforme* to develop fermented soybean beverages [11]. The total free amino acid content and composition of the soybean beverage after fermentation by *N. aurantialba* increased. The essential amino acid levels of FSB increased by 47.4% compared to that of UFSB, along with the production of cysteine and proline, which were absent from UFSB, implying that fermentation of soybean beverages by *N. aurantialba* increased the amino acid levels (Table S1). According to Yang et al., the higher content of free amino acids produced in fermented soymilk is due to the high activity of proteolytic enzymes in *Grifola frondosa*, which could be used to digest soybean proteins, suggesting that *N. aurantialba* may also contain high proteolytic enzyme activity that could be used to digest soybean proteins [31]. In addition, the increased levels of free amino acids meant that soy beverages were more easily absorbed after fermentation [1].

Reducing sugar and total sugar concentrations were compared between the two fermented beverages, and the levels of total and reducing sugar concentrations in the FSB were significantly higher than in the UFSB. The changes in reducing sugars were mainly due to the exogenous addition of glucose at 40 g/L for fungal growth. The increase in total sugar levels was mainly due to *N. aurantialba* metabolism, which utilized a large amount of glucose as a matrix to generate 8.68 g/L of NAPS (Table 1 and Figure S1). Because of their unique health properties, polysaccharides are frequently used to improve the health value of beverages [49]. In *Caenorhabditis elegans*, a beverage rich in polysaccharides of *Cyclocarya paliurus* can protect against oxidative stress and reduce fat deposition without affecting critical physiological activities [50]. Furthermore, frequent ingestion of yogurt containing hallabong peel polysaccharide boosts natural killer NK cell capacity while decreasing proinflammatory cytokine levels [51]. As a result, after *N. aurantialba* fermentation, the bioactivity of soy drinks may increase.

The content of total acid is also one of the important factors in evaluating the composition of beverages. The total acid level in the soy beverage was 0.54 g/kg after fermentation, which showed an increase of 37% compared with its unfermented counterparts; this indicated the decreased pH of the FSB. This means that the FSB had the characteristics of a “sour” lactic acid bacteria beverage. Lactic acid bacteria beverages tend to all have a lower pH due to the large amount of lactic acid produced by fermentation [40]. Furthermore, *Ganoderma lucidum* fermentation reduced the pH of pumpkin juice from 7 to 4, giving the drink a sour flavor [52].

In addition, the reason that *N. aurantialba* presented acidity might be because of the production of uronic acid as well as other organic acids by bacteriophage metabolism.

Phenolic compounds and flavonoids, such as rutin, catechin, and naringin, are widely found in raw materials from plants, microbial, and other sources, and are known to have important antioxidant activities, so we examined the contents of total polyphenols and flavonoids in UFSB and FSB [32]. In FSB and UFSB, total polyphenol content was 2313.3 mg/L and 1285.5 mg/L, respectively. Additionally, the total flavonoid content in FSB and UFSB was 1040.2 mg/L and 603.44 mg/L, respectively. Thus, the total antioxidant content of FSB was higher than that of UFSB, which means that FSB has potential as a functional food. Because phenolics are the major antioxidant components in mushrooms, and their contents can be used to evaluate the antioxidant capacity of beverages to some extent, it is important to detect the presence of phenolics in FSB. Islam et al. found that the content of polyphenols and flavonoids in *N. aurantialba* was 0.80 mg GAE/g and 0.13 mg GAE/g, respectively [53]. The kinds of phenolics might include homogenous acid, protocatechuic acid, and p-hydroxybenzoic acid, for example, and the findings could be evaluated using LC-MS due to the non-specificity of the Folin–Ciocalteu reagent [21]. 

### 3.2. Antioxidant Capacity of Soy Beverages

**Figure 1:**
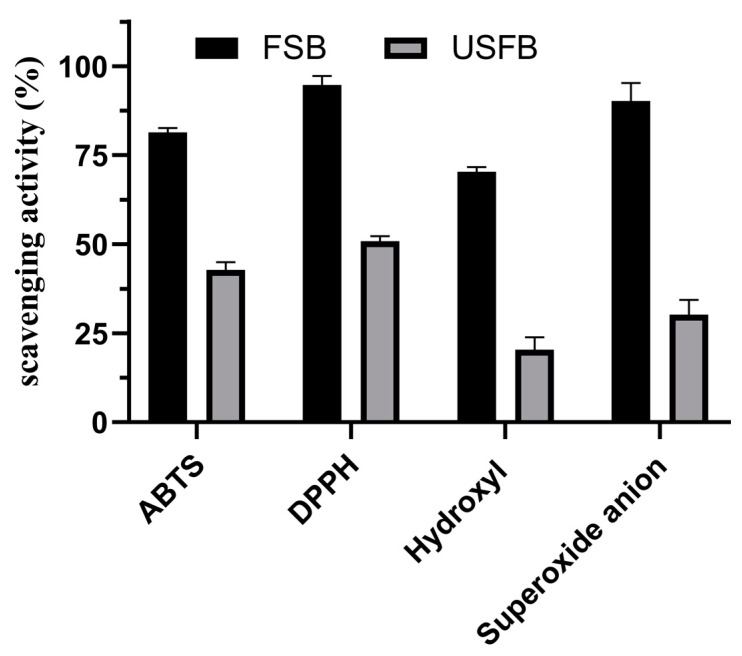
Antioxidant capacity of soy beverage with *N. aurantialba*.

Antioxidant capacity is an important indicator often used to assess whether fermented beverages have health care value [54]. Figure 1 displays the antioxidant capacity of FSB. As no single method can be used to describe the overall antioxidant capacity of a sample, four antioxidant detection techniques were used to examine the antioxidant capacities of FSB and UFSB: ABTS radical scavenging, DPPH radical scavenging, OH radical scavenging, and superoxide anion radical scavenging. The ABTS radical scavenging capacity of FSB was 81.4%, while that of UFSB was 42.8%; it increased in FSB by 90.1%. The DPPH radical scavenging capacity of FSB was 94.75%, while that of UFSB was 50.88%. It increased in FSB by 86.22%. The hydroxyl radical scavenging capacity of FSB was 70.35%, while that of UFSB was 20.35%; after fermentation, this value increased by 71.07%. FSB had a 90.22% superoxide anion radical scavenging capacity, whereas for UFSB it was 30.28%; thus, fermentation increased it 2.31-fold. The free radical scavenging capacity and total antioxidant capacity of FSB were significantly higher than those of UFSB, possibly because the total polyphenol and flavonoid levels, which exhibited antioxidant activity, of FSB were 1.8 and 1.7 times higher than those of UFSB, respectively. In addition, NAPS, a natural macromolecular polysaccharide, also has a certain antioxidant capacity [17,55]. Based on the antioxidant analysis, it was not surprising that the antioxidant activity of soybean beverage fermented by *N. aurantialba* increased significantly. These differences may be attributed to the reaction rate of free radical sources to polysaccharides, total phenols and flavonoids, and other active substances in FSB. Therefore, FSB with effective antioxidant capacity may be an effective health drink for humans.

### 3.3. Volatile Fingerprints of Soybean Beverage by HS-GC-IMS

**Figure 2:**
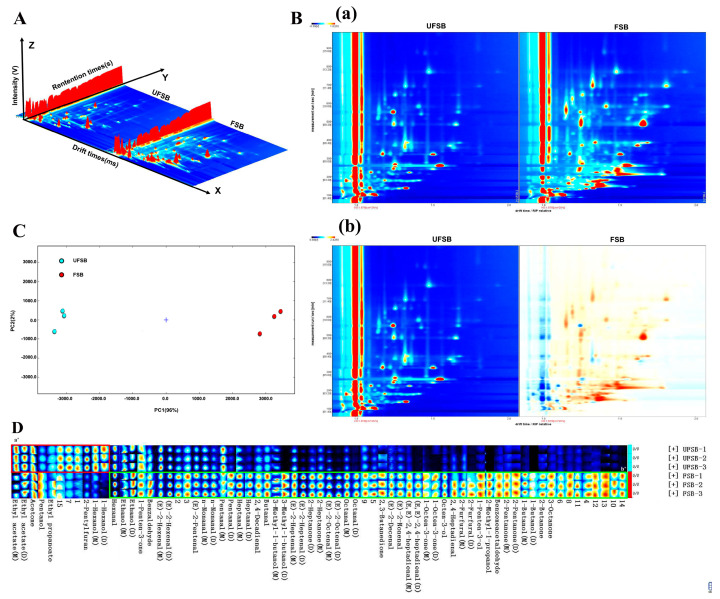
The volatile organic compounds analysis in soy beverage by HS-GC-IMS. (**A**) 3D-topographic features of samples, (**B**) 2D-topographic top view plot of samples (**a**): normal top view plot, (**b**): 2D-topographic top view plot deduction), (**C**) PCA score of samples, and (**D**) fingerprint spectra of samples.

HS-GC-IMS was used to investigate flavor profiles in the soybean beverages before and after fermentation, and the data is presented as 3D visualized topography (Figure 2A) and 2D top view plots (Figure 2B). The results showed that there were large differences in signal properties and intensities between FSB and UFSB. The first two principal components accounted for 96% and 2% of the total variance, respectively (Figure 2C). In addition, the PCA results showed that FSB and UFSB occupied relatively independent spaces in the profiles, implying that their flavor profiles were quite different. To gain more insight into the specific types of compounds identified, the volatile fingerprints of FSB were successfully established (Figure 2D). From the volatile fingerprint, we obtained a color, and a darker color indicated a higher intensity. In addition, some single compounds with high concentrations or proton affinities produced more than one signal, corresponding to dimers and trimers, which were retained at similar times but migrated at different times [56]. The volatile fingerprint information indicated that 69 typical aromas were tentatively identified in the FSB, with some components exhibiting two peaks corresponding to monomeric and dimeric forms, and the dimers being eluted after the monomers. The compounds in region a′ of Figure 2D, including 2-pentylfuran, 1-hexanol, (E)-2-hexenal, hexanal, pentanal, and ethyl acetate, were abundant in the UFSB sample. In contrast, the signal intensities of volatile compounds in region b′ consisting of 1-octen-3-ol, 3-octanone, 1-octen-3-one (M), and 1-octen-3-one (D), significantly increased after fermentation. 

The peak intensities and relative contents of VOCs in the soy beverages are shown in Table 2 and Table S2. These compounds were classified into six categories, including 12 alcohols, 26 aldehydes, 3 esters, 11 ketones, 1 furan, and 15 unidentified compounds. Hexanal, regarded as one of the most important contributors to the beany flavor of soybean, decreased significantly in both peak intensity and relative content after fermentation [9]. In addition, other substances that were also regarded as the main sources of the beany flavor, such as (E)-2-hexenal, pentanal, 2-pentylfuran, 1-hexanol, and other compounds, also showed a significant decrease in signal intensity. To date, little data is available on the mechanism by which basidiomycetes degrade or adsorb beany flavor compounds such as hexanal, hexanol, and (E)-2-hexenal [57]. Based on studies on using Bifidobacterium to ferment soymilk, it can be speculated that the key enzymes needed to oxidize aldehydes to acids seem to be aldehyde dehydrogenase and aldehyde dehydrogenase [58].

In addition, the generation of more volatile compounds during fermentation may enhance some positive aroma properties in soy beverages, thereby eliminating or masking the beany flavor of soybeans [6,12,59]. Because of their low odor threshold and strong odor properties, aldehydes were the most detected compounds and important contributors to the aroma of FSB [60]. Substances such as pentanal (fruit, berry), benzeneacetaldehyde (green, sweet, cocoa), and (E)-2-pentenal (green, fruity) showed a greater increase in signal intensities in soy beverages after fermentation. Two compounds, pentanal and benzeneacetaldehyde, which are considered to be two pleasant odors of the fruiting body of *Tricholoma matsutake*, are mainly produced by the oxidation of polyunsaturated fatty acid double bonds [37,61]. Ketones are generated by polyunsaturated fatty acid oxidation, the Maillard reaction, amino acid degradation, or microbial oxidation [62]. The signal intensities of 1-octen-3-one (D), 1-octen-3-one (M), and 3-octanone in FSB increased by 8.67-, 3.71-, and 1.26-fold, respectively, compared with those of UFSB. Moreover, in addition to the large increases in signal intensities of 1-octen-3-one and 3-octanone with the mushroom odor, significant increases in the signal intensities of 2-pentanone, 2-butanone, 2-heptanone (M), 2-heptanone (D), 2,3-butanedione, and acetone with a fruity odor were also observed. Compared with corresponding ketones and aldehydes, the threshold of alcohols is higher and can be divided into saturated and unsaturated alcohols. Unsaturated alcohols have low thresholds and distinctive odors [37]. Among the alcohols that should be noted is 1-octen-3-ol, which is known as the mushroom alcohol and T. matsutake alcohol and is considered to be one of the major substances responsible for the flavor of mushrooms [37,61]. Fermented soy beverages had a 2.78-fold increase in 1-octen-3-ol compared to UFSB. In addition, the signal intensities of some substances with an alcoholic flavor, such as 2-methyl-1-propanol, 1-pentanol, 3-methyl-1-butanol (M), and 3-methyl-1-butanol (D), were also significantly increased, but it was speculated that they had limited influence on the overall flavor because of their higher odor thresholds. 

It must be emphasized that a series of eight carbon compounds are key contributors to the mushroom flavor, including (E)-2-octenal, 1-octen-3-one, 1-octen-3-ol, and 3-octanone (Table 2 and Table S2). These substances are considered typical volatile compounds in edible mushrooms and are major contributors to the unique flavor of mushrooms, probably produced by the metabolism of fatty acids by edible mushrooms [63]. This means that FSB is highly likely to have a pleasant mushroom aroma.

### 3.4. Volatile Fingerprints of Soybean Beverage by HS-SPME-GC-MS

**Figure 3:**
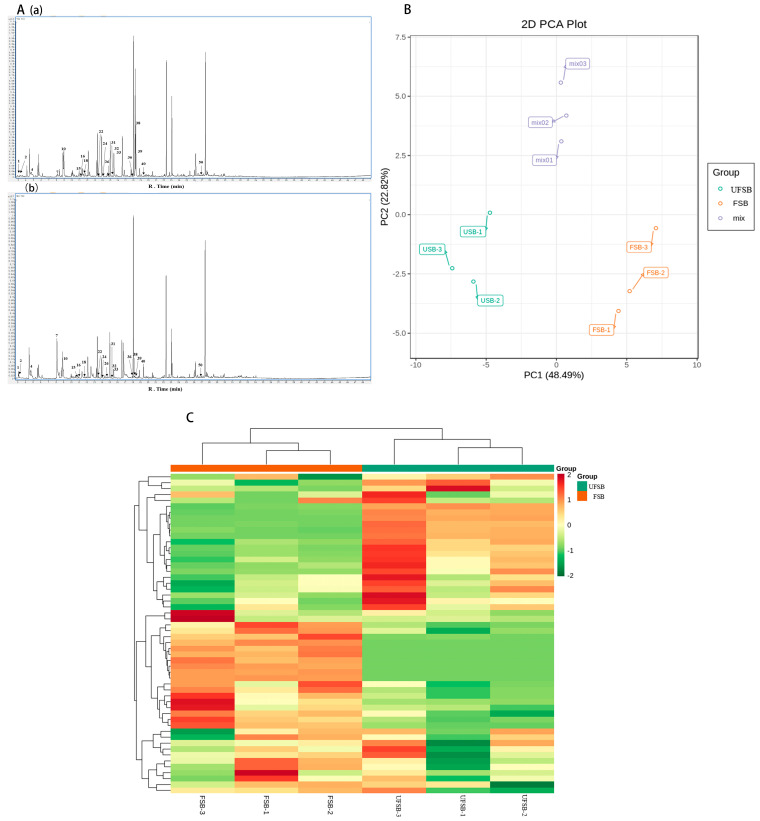
The volatile organic compounds analysis in soy beverage by HS-SPME-GC-MS. (**A**) GC-MS total ion current chromatograms of (**a**) the unfermented soy beverage and (**b**) the fermented soy beverage with *N. aurantialba*, (**B**) PCA score of samples, (**C**) heat map visualization of samples.

To further evaluate the effects of *N. aurantialba* fermentation on the flavor of soybean beverages, HS-SPME-GC-MS was used to analyze the VOCs in the samples. Similar to the HS-GC-IMS results, the number and area of VOCs peaks changed with fermentation, indicating that fermentation altered VOCs formation and, consequently, the flavor of the soy beverage (Figure 3A). As shown in Figure 3B, the results of PCA showed that the first two principal components accounted for 48.49% and 22.8% of the total variance, and FSB and UFSB occupied relatively independent spaces in the profiles, which was similar to the results of GC-IMS, further illustrating that the flavor substances in the samples were different.

In UFSB and FSB, ten classes 49 and ten classes 54 recognized volatile chemicals were found, including heterocyclic compounds, aromatics, alkanes, alkenes, alcohols, aldehydes, ester ketones, and terpenoids (Table 3 and Table S3, Figure 3C). The most detected compounds were heterocycles, and the peak areas of the four heterocycles, including oxetane, 3-(1-methylethyl)-, thiophene, 2-pentyl-, thiophene, 2-hexyl-, 1-pentanone, and 1-(2-furanyl)-, increased significantly. All compounds, except for oxetane, 3-(1-methylethyl), which had no odor description, were Maillard reaction-derived compounds with an odor description of “fruity, sweet.” Researchers have found that these substances are characteristic aromas in meats such as pork, beef, and lamb, and may also be responsible for the meat flavor of some mushrooms [64]. Interestingly, thiophene, 2-pentyl-, was a derivative of cysteine, which was produced from a soy beverage after fermentation by *N. aurantialba* (Table S1).

The peak areas of the two furan heterocycles, 2-pentylfuran and (E)-2-(1-pentenyl)-furan, decreased significantly, and they were one of the browning reaction’s volatiles (Table 3 and Table S3). However, their flavor was described as “green and fatty.” A decrease in their levels may improve the beany flavor of the soybean beverage. Under the action of microorganisms, benzaldehyde, a compound produced by the degradation of phenylalanine under the action of microorganisms, showed increased levels following fermentation, which was consistent with the results of the GC-IMS [65]. In addition, the levels of various VOCs, such as (E, E)-2,4-heptadienal, 2-octenal, and 4-ethyl-benzaldehyde, significantly decreased, possibly contributing to the reduction in the herbaceous and green aroma of soy beverages caused by excessive amounts of such compounds.

In mushroom-fermented beverages, 1-octen-3-ol increased 2.5-fold as the primary representative of alcoholic aroma substances, which is compatible with the GC-IMS findings. The signal intensities of these esters (2 (3H)-furanone, dihydro-5-pentyl, octanoic acid, ethyl ester, and methyl salicylate) were substantially greater in FSB than in UFSB, showing that *N. aurantialba* contributes to the improved fragrance intensity and complexity of the soybean beverages.

### 3.5. Analysis of the Relative Odor Activity Values

To evaluate the contribution of different compounds to the odor of soy beverages, the ROAV was determined and calculated to assess the overall odor levels (Table 4).

From GC-IMS analysis, the main volatile flavor compounds in the control group were octanal, 1-octen-3-one, (E)-2-nonenal, 2,4-decadienal, hexanal, 1-octen-3-ol, 2-pentylfuran, and a (E)-2-octenal), whereas those in the FSB group were 1-octen-3-one (mutagen), and others were octanal, (E)-2-nonenal, in that order, compounds such as 2,4-decadienal, and 1-octen-3-ol. After fermentation, the ROAV values of the beany flavor substances, mainly aldehydes, such as hexanal, octanal, 2,4-decadiena, and (E)-2-nonenal, decreased by 80%, 20%, 60%, and 40%, respectively.

From the GC-MS analysis, the main volatile flavor components of the control group were in the order of compounds such as (E, E)-2,4-decadienal (fatty, green), 2-pentylfuran (green), and 2-octenal (fatty, green). After 5 d of fermentation, the FSB group had a total of eight VOCs with high ROAV (ROAV > 1), including 1-octen-3-ol, (E, E)-2,4-decadienal, 2-octenal, 2 (3H)-furanone, dihydro-5-pentyl-, octanoic acid, ethyl ester, and 2-pentylfuran. The study by Aschemann Witzel et al. similarly demonstrated the degradation of green VOCs, such as 2,4-decadienal, in soybean beverages fermented using basidiomycetes to improve the flavor of soybean beverages. However, the mechanism of (E, E)-2,4-decadienal degradation by basidiomycetes is yet to be fully understood [11]. In addition, the strongest contributor to the typical aroma of FSB fermented by *B. piriformis* was 1-octen-3-one, which is similar to our findings that 1-octen-3-one provided a pleasant characteristic odor following fermentation by basidiomycetes [11].

Thus, although the detection sensitivity and VOCs were distinguishable between GC-IMS and GC-MS, the results of these two methods collectively indicated, to some extent, that the beany flavor substances of soybean beverages decreased after fermentation, and that the mushroom, fruity, and sweet aromas dominated.

### 3.6. Analysis of the Relative Odor Activity Values

To evaluate the contribution of different compounds to the odor of soy beverages, the ROAV was determined and calculated to assess the overall odor levels.

From GC-IMS analysis, the main volatile flavor compounds in the control group were octanal, 1-octen-3-one, (E)-2-nonenal, 2,4-decadienal, hexanal, 1-octen-3-ol, 2-pentylfuran, a(E)-2-octenal, whereas those in the FSB group were 1-octen-3-one (mutagen), and others were octanal, (E)-2-nonenal, in that order, and compounds such as 2,4-decadienal and 1-octen-3-ol. After fermentation, the ROAV values of the beany flavor substances, mainly aldehydes, such as hexanal, octanal, 2,4-decadiena, and (E)-2-nonenal, decreased by 80%, 20%, 60%, and 40%, respectively.

From the GC-MS analysis, the main volatile flavor components of the control group were in the order of compounds such as (E, E)-2,4-decadienal (fatty, green), 2-pentylfuran (green), and 2-octenal (fatty, green). After 5 d of fermentation, the FSB group had a total of eight VOCs with high ROAV (ROAV > 1), including 1-octen-3-ol, (E, E)-2,4-decadienal, 2-octenal, 2 (3H)-furanone, dihydro-5-pentyl -, octanoic acid, ethyl ester, and 2-pentylfuran. The study by Aschemann Witzel et al. similarly demonstrated the degradation of green VOCs, such as 2,4-decadienal, in soybean beverages fermented using basidiomycetes to improve the flavor of soybean beverages [11]. However, the mechanism of (E, E)-2,4-decadienal degradation by basidiomycetes is yet to be fully understood. In addition, the strongest contributor to the typical aroma of FSB fermented by *B. piriformis* was 1-octen-3-one, which is similar to our findings that 1-octen-3-one provided a pleasant characteristic odor following fermentation by basidiomycetes [11].

Thus, although the detection sensitivity and VOCs were distinguishable between GC-IMS and GC-MS, the results of these two methods collectively indicated, to some extent, that the beany flavor substances of soybean beverages decreased after fermentation, and that the mushroom, fruity, and sweet aromas dominated.

### 3.7. Sensory Property of Soy Beverages

**Figure 4:**
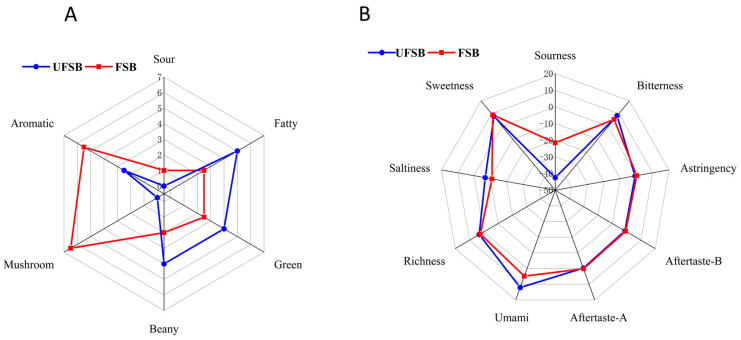
Radar chart of FSB and UFSB. (**A**) Sensory property of soy beverages, (**B**) electronic tongue measurement of soy beverages.

To evaluate the flavor of FSB, we ran sensory analyses. The results of the two samples are shown in Figure 4A. Sensory evaluation showed that the characteristic odor of UFSB was beany, green, and fatty. The characteristic flavors of FSB were mushroom and aromatic. This is consistent with the results of the ROAV analysis. In addition, the FSB similarly exhibited an acidic note, which consisted of the low pH of the FSB. Overall, FSB had a softer, consumer-satisfying odor.

### 3.8. Electronic Tongue Measurement

A radar plot is shown in Figure 4B, showing the results of our investigations into the compounds contributing to the umami, bitter, astringent, tangy, sour, and salty notes in soy beverages. It is evident from the figure that there were significant differences between the two groups of samples, with a large rise in sourness, a small decrease in both bitter and bitter echoes, a smaller rise in sweet and astringency, and a slight decrease in umami and richness in FSB compared to UFSB. It is known that fermentation decreases the bitter taste of soy beverages but increases sourness while maintaining the fresh and sweet taste of natural soybean. Thus, FSB has potential in terms of its success in the fermented soybean beverage industry. The higher levels of glucose in the FSB could have masked the bitterness [66]. In future research, we would like to investigate whether we can optimize the glucose supply for initial fermentation such that the strain utilizes all of the glucose, thus resolving the issue where the difference in glucose levels in the sample influences sensory evaluation. Microbial fermentation has been reported to alter the taste of beverages to some extent [12,59]. For example, lactic acid bacteria are used to ferment ginkgo juice; acetic acid bacteria are used to ferment kombucha tea; yeasts are used to ferment juices such as pomegranate, apple, and strawberry juices, changing their flavor profiles [67,68,69]. Despite the fact that basidiomycete-fermented beverages have not been substantially explored, Zhao et al. found that following *G. lucidum* fermentation, the flavor of pumpkin juice transforms from a disagreeable “stewing” to a pleasant “fruity and flowery” note [52]. In general, changes in the flavor profiles of microbial-fermented beverages are closely associated with the metabolites of the strains themselves. For example, *L. plantarum* was used to ferment *Z. jujuba* juice, and the resulting juice was mainly characterized by acidic notes with decreased bitter and astringent notes due to the production of large amounts of lactic acid, aromatic compounds, and sulfur organics when metabolized [70].

## 4. Conclusions

In this study, we investigated the feasibility of fermented soybean beverages to enrich the diversity of plant-based products. FSB was prepared from soybean as a raw material, using *N. aurantialba* as a species. Soy beverages become more stable because of the increase in Zeta potential, particle size, and viscosity. The nutrient composition was increased by the fermentation of *N. aurantialba*. The antioxidant activity of soybean beverages significantly increased after 5 d of fermentation. By reducing the content of beany substances such as hexanal and increasing the content of 1-octen-3-ol, the aroma of soybean beverage fermented by *N. aurantialba* changed from “beany, green, fatty” to “mushroom and aromatic”. FSB not only reduces bitterness but also greatly increases sourness while maintaining the fresh and sweet taste of UFSB. The results of this study indicate that the soy beverage fermented by *N. aurantialba* could not only improve the health characteristics of soy beverages but also reduce the beany taste of soy beverages, which means that it has the potential to be marketed. However, the degradation pathways of *N. aurantialba* for these beany-flavored substances should be investigated in future studies using isotope labeling experiments. In addition, multi-omics technologies such as transcriptomics, metabolomics, and proteomics can be used to uncover the underlying causes of how *N. aurantialba* fermentation impacts volatile chemicals in soymilk and to better understand the VOCs changing mechanism. More research into these elements will help the development of basidiomycete fermentation technologies in the food industry.

## Figures and Tables

**Table 1:** Physicochemical parameters and nutritional ingredients of the unfermented and fermented soy beverages with *N. aurantialba*.

Parameter		FSB	UFSB
particle size distribution (nm)	D_50_	926 ± 32 ^A^	198 ± 20 ^B^
Zeta potential (mv)		−32.5 ± 2.10 ^A^	−12.4 ± 2.40 ^B^
pH		4.00 ± 0.03 ^A^	6.50 ± 0.02 ^B^
appearance		milk white, faint yellow	milk white, faint yellow
viscosity (mPa·s)		804 ± 5.6 ^A^	3.40 ± 0.5 ^B^
L*a*b color space	L*	78.5 ± 0.58 ^A^	77.6 ± 1.05 ^A^
	a*	−1.41 ± 0.02 ^A^	−1.21 ± 0.13 ^B^
	b*	8.32 ± 0.09 ^A^	7.13 ± 0.09 ^B^
protein (g/kg)		17.0 ± 1.3 ^A^	28.0 ± 1.5 ^B^
fatty acid (g/kg)		20.0 ± 0.43 ^A^	21.2 ± 6.4 ^A^
total sugar (g/L)		13.1 ± 0.21 ^A^	2.94 ± 0.18 ^B^
reducing sugar (g/L)		4.28 ± 0.41 ^A^	1.03 ± 0.28 ^B^
glucose (g/L)		3.12 ± 0.12 ^A^	0.17 ± 0.05 ^B^
dietary fiber (%)		4.20 ± 0.50 ^A^	4.60 ± 0.80 ^A^
total acid (g/kg)		5.40 ± 0.06 ^A^	3.40 ± 0.04 ^B^
total amino acids (μg/mL)		32.8 ± 0.008 ^A^	32.0 ± 0.011 ^B^
total polyphenol (mg/L)		2313 ± 6.48 ^A^	1285 ± 8.24 ^B^
total flavonoid (mg/L)		1080 ± 3.59 ^A^	603 ± 2.65 ^B^
NAPS (g/L)		8.68 ± 0.12 ^A^	0 ^B^

Each value is expressed as mean ± SD (*n* = 3); means with different capital letters within a row indicate significant differences (*p* < 0.05); L* value represents the lightness of the sample, and a* value and b* value indicate the redness and yellowness, respectively.

**Table 2:** Identification of the volatile compounds in fermented soy beverage by HS-GC-IMS.

Count	Compounds	RI	Rt (s)	Dt (a.u.)	Odor Description	Relative Amount (%)	
						UFSB	FSB
		Aldehydes					
1	2,4-Decadienal	1285	1352	1.42	fatty green	1.20 ± 0.011 ^A^	1.03 ± 0.054 ^B^
2	(E)-2-Decenal (M)	1233	1148	1.48	fatty, fish, hay	0.520 ± 0.052 ^A^	0.530 ± 0.025 ^A^
3	(E)-2-Nonenal (D)	1147	879	1.41	fatty green	0.280 ± 0.052 ^A^	0.330 ± 0.030 ^A^
4	n-Nonanal (M)	1106	772	1.48	rose fresh fruity	2.33 ± 0.080 ^A^	1.76 ± 0.057 ^B^
5	n-Nonanal (D)	1106	772	1.94		0.310 ± 0.035 ^A^	0.280 ± 0.016 ^A^
6	(E)-2-Octenal (M)	1072	695	1.34	fatty green	2.85 ± 0.430 ^A^	4.44 ± 0.239 ^B^
7	(E)-2-Octenal (D)	1072	694	1.81		0.480 ± 0.023 ^A^	1.93 ± 0.205 ^B^
8	(E, E)-2,4-heptadienal (M)	1036	621	1.19	fatty green	0.620 ± 0.036 ^A^	1.06 ± 0.095 ^B^
9	(E, E)-2,4-heptadienal (D)	1035	619	1.61		0.090 ± 0.002 ^A^	0.370 ± 0.032 ^B^
10	2,4-Heptadienal	1019	590	1.20	green fruity	0.330 ± 0.092 ^A^	0.380 ± 0.124 ^A^
11	Octanal (M)	1015	581	1.42	green fatty	1.20 ± 0.044 ^A^	1.77 ± 0.029 ^B^
12	Octanal (D)	1014	580	1.82		0.210 ± 0.021 ^A^	0.650 ± 0.037 ^B^
18	(E)-2-Heptenal (M)	964	485	1.26	green fatty	3.59 ± 0.245 ^A^	4.37 ± 0.068 ^B^
19	(E)-2-Heptenal (D)	964	484	1.66		2.34 ± 0.088 ^A^	9.72 ± 0.031 ^B^
20	Benzaldehyde	981	518	1.15	bitter almond, sweet cherry	0.900 ± 0.159 ^A^	0.650 ± 0.128 ^A^
21	Heptanal (M)	905	387	1.36	fatty green	1.45 ± 0.118 ^A^	1.33 ± 0.020 ^B^
22	Heptanal (D)	904	385	1.69		0.580 ± 0.023 ^A^	1.27 ± 0.035 ^B^
27	(E)-2-hexenal (M)	858	325	1.18	green fatty	3.55 ± 0.057 ^A^	1.59 ± 0.037 ^B^
28	(E)-2-hexenal (D)	855	322	1.51		3.58 ± 0.217 ^A^	2.88 ± 0.062 ^B^
29	2-Furfural (M)	833	298	1.09	sweet woody almond	0.260 ± 0.028 ^A^	0.260 ± 0.100 ^A^
30	2-Furfural (D)	831	295	1.33		0.040 ± 0.0003 ^A^	0.130 ± 0.035 ^B^
31	hexanal	796	260	1.55	green fatty	17.3 ± 0.708 ^A^	7.38 ± 0.039 ^B^
32	(E)-2-pentenal	756	224	1.36	green fruity	1.27 ± 0.095 ^A^	1.05 ± 0.002 ^B^
33	pentanal (M)	699	180	1.19	fruity berry	1.49 ± 0.074 ^A^	0.620 ± 0.030 ^B^
34	pentanal (D)	701	182	1.43		0.730 ± 0.02 ^A^	1.70 ± 0.109 ^B^
38	butanal	605	135	1.29	pungent cocoa green	0.890 ± 0.039 ^A^	0.900 ± 0.021 ^A^
54	benzeneacetaldehyde	1071	693	1.26	green sweet cocoa	0.230 ± 0.004 ^A^	0.260 ± 0.018 ^A^
13	1-Octen-3-ol	998	552	1.16	mushroom	2.06 ± 0.072 ^A^	3.48 ± 0.082 ^B^
25	1-hexanol (M)	886	359	1.32	fruity alcoholic sweet green	2.55 ± 0.032 ^A^	0.500 ± 0.065 ^B^
26	1-hexanol (D)	884	357	1.64		0.600 ± 0.094 ^A^	0.080 ± 0.016 ^B^
35	2-Methyl-1-propanol	632	146	1.37	ethereal winey cortex	0.250 ± 0.012 ^A^	5.49 ± 0.124 ^B^
40	ethanol (M)	483	94.5	1.05	strong alcoholic	10.5 ± 0.367 ^A^	5.51 ± 0.062 ^B^
41	ethanol (D)	486	95.2	1.14		1.99 ± 0.251 ^A^	1.74 ± 0.080 ^A^
50	1-butanol (M)	668	163	1.17	sweet balsam whiskey	0.430 ± 0.042 ^A^	1.10 ± 0.023 ^B^
51	1-butanol (D)	667	162	1.39		0.040 ± 0.004 ^A^	1.53 ± 0.017 ^B^
52	3-Methyl-1-butanol (M)	744	214	1.24	alcoholic fruity	1.35 ± 0.059 ^A^	0.990 ± 0.030 ^B^
53	3-Methyl-1-butanol (D)	745	215	1.50		0.410 ± 0.023 ^A^	7.70 ± 0.062 ^B^
43	1-penten-3-ol	692	175	1.34	fruity green	0.450 ± 0.007 ^A^	0.900 ± 0.004 ^B^
44	1-Pentanol	777	243	1.51	sweet balsam	0.650 ± 0.044 ^A^	0.660 ± 0.011 ^A^
14	2-Pentylfuran	999	553	1.25	green beany vegetable	5.28 ± 0.040 ^A^	1.09 ± 0.132 ^B^
15	3-Octanone	993	543	1.31	sweet mushroom	0.170 ± 0.020 ^A^	0.200 ± 0.002 ^A^
16	1-Octen-3-one (M)	987	531	1.27	mushroom	0.150 ± 0.007 ^A^	0.370 ± 0.040 ^B^
17	1-Octen-3-one (D)	986	528	1.68		0.120 ± 0.076 ^A^	0.590 ± 0.050 ^B^
23	2-heptanone (M)	895	372	1.26	fruity sweet	0.750 ± 0.017 ^A^	0.910 ± 0.008 ^B^
24	2-heptanone (D)	896	373	1.63		1.14 ± 0.106 ^A^	1.79 ± 0.037 ^B^
39	2-Butanone	592	130	1.24	fruity	0.200 ± 0.042 ^A^	0.330 ± 0.014 ^B^
42	Acetone	512	103	1.13	apple pear	7.75 ± 0.893 ^A^	5.01 ± 0.168 ^B^
43	1-penten-3-one	689	173	1.08	pungent peppery onion	0.250 ± 0.039 ^A^	0.100 ± 0.003 ^B^
47	2,3-butanedione	591	130	1.17	sweet creamy	0.210 ± 0.049 ^A^	0.270 ± 0.008 ^A^
48	2-pentanone (M)	687	172	1.12	sweet fruity	0.080 ± 0.006 ^A^	0.110 ± 0.001 ^B^
49	2-pentanone (D)	691	175	1.36		0.480 ± 0.002 ^A^	0.820 ± 0.011 ^B^
36	ethyl acetate (M)	606	136	1.10	fruity sweet green	3.20 ± 0.154 ^A^	0.770 ± 0.022 ^B^
37	ethyl acetate (D)	612	138	1.33		5.60 ± 0.213 ^A^	3.02 ± 0.054 ^B^
45	ethyl propanoate	710	188	1.15	sweet fruity	0.120 ± 0.014 ^A^	0.050 ± 0.021 ^B^

Note: Rt: Represents the retention time in the capillary GC column; RI: represents the retention index calculated using n-ketones C4–C9 as external standard on FS-SE-54-CB-1 column; each value is expressed as mean ± SD (*n* = 3); “(M)”, Monomer; “(D)”, Dime; for Relative amount: means with different capital letters within a row indicate significant differences (*p* < 0.05); relative amount, the percentage of each compound peak intensity to total peak intensity of all compounds.

**Table 3:** Identification of the volatile compounds in fermented soy beverage by HS-SPME-GC-MS.

No.	RT	Compounds	RI	NIST_RI	Odor Description	Peak Intensity	
						UFSB	FSB
Heterocyclic compound							
1	3.06	Oxetane, 3-(1-methylethyl)-	718.12	664	-	ND	1,621,445 ± 196,936
10	8.96	2-pentylfuran	987.89	993	green earthy beany	6,498,125 ± 534,065 ^a^	712,224 ± 128,211 ^b^
14	10.8	(E)-2-(1-pentenyl)-furan	1051.11	1048	roasted	186,144 ± 31,660 ^a^	125,155 ± 7559 ^b^
22	13.5	Thiophene, 2-pentyl-	1143.43	1090	fruit, sweet	ND	628,882 ± 20,192
26	14.78	Thiophene, 2-hexyl-	1187.4	1277	floral fruity gassy	ND	251,010 ± 32,886
36	17.91	1-Pentanone, 1-(2-furanyl)-	1299.53	1176	sweet caramel	184,692 ± 27,820 ^a^	557,517 ± 87,545 ^b^
Alcohol							
8	8.67	1-Octen-3-ol	977.65	982	mushroom	984,538 ± 121,842 ^a^	3,477,799 ± 1,627,475 ^b^
Aldehyde							
7	8.11	Benzaldehyde	957.34	960	bitter almond, sweet cherry	1,346,850 ± 149,896 ^a^	16,021,725 ± 803,440 ^b^
11	9.58	(E, E)-2,4-heptadienal	1009.64	1011	fatty green	458,123 ± 83,423 ^a^	292,071 ± 65,426 ^b^
15	10.95	2-octenal	1056.04	1060	fatty green herbal	510,065 ± 53,147 ^a^	150,411 ± 12,114 ^b^
24	14.01	Benzaldehyde, 4-ethyl-	1160.8	1171	bitter almond	316,945 ± 43,024 ^a^	72,065 ± 1910 ^b^
39	18.34	2,4-Decadienal, (E,E)-	1315.79	1317	fatty green	1,186,788 ± 64,214 ^a^	57,711 ± 9133 ^b^
Ester							
27	14.85	Methyl salicylate	1189.63	1234	wintergreen mint	134,718 ± 47,046 ^a^	80,900 ± 8110 ^b^
28	15	Octanoic acid, ethyl ester	1194.8	1198	sweet fruity	4,720,782 ± 406,796 ^a^	7,761,488 ± 1,037,991 ^b^
40	19.39	2(3H)-Furanone, dihydro-5-pentyl-	1355.43	1366	coconut creamy sweet buttery	120,699 ± 23,131 ^a^	5,154,713 ± 83,079 ^b^

Note: Each value is expressed as mean ± SD (*n* = 3); for peak intensity: means with different lowercase letters within a row indicate significant differences (*p* < 0.05); ND, not detected; “-”, not described.

**Table 4:** Relative odor activity values (ROAV) and odor description of volatile compounds of soy beverage.

CAS.	Compounds	Odor Thresholds (mg/kg)	ROAV				Odor Description
			GC-IMS		GC-MS		
			UFSB	FSB	UFSB	FSB	
100-52-7	Benzaldehyde	0.3	<1	<1	-	-	bitter almond, sweet cherry
110-62-3	Pentanal	0.012	1.0	<1	-	-	fruity berry
111-71-7	Heptanal	0.01	1.2	<1	-	-	fatty green
122-78-1	Benzeneacetaldehyde	0.004	<1	<1	-	-	green sweet cocoa
123-72-8	Butanal	0.00526	1.4	<1	-	-	pungent cocoa green
124-13-0	Octanal	0.0001	100.0	75.4	-	-	green fatty
124-19-6	n-Nonanal	0.0035	5.5	2.2	-	-	rose fresh fruity
1576-87-0	(E)-2-Pentenal	0.98	<1	<1	-	-	green fruity
18829-55-5	(E)-2-Heptenal	0.013	2.3	1.4	-	-	green fatty
18829-56-6	(E)-2-Nonenal	0.000065	35.9	21.9	-	-	fatty green
2363-88-4	2,4-Decadienal	0.0003	33.3	14.7	-	-	fatty green
2363-89-5	2-octenal	0.0002	-	-	6.4	32.6	fatty green herbal
4313-03-5	(E, E)-2,4-heptadienal	0.0154	<1	<1	<1	<1	fatty green
4748-78-1	Benzaldehyde, 4-ethyl-	0.12323	-	-	<1	<1	bitter almond
5910-85-0	2,4-Heptadienal	0.15	<1	<1	-	-	green fruity
66-25-1	Hexanal	0.0045	31.9	7.0	-	-	green fatty
6728-26-3	(E)-2-Hexenal	0.04	<1	<1	-	-	green fatty
3913-81-3	(E)-2-Decenal	0.25	<1	<1	-	-	fatty, fish, hay
25152-84-5	2,4-Decadienal, (E,E)-	0.000027	-	-	100.0	84.9	fatty green
2548-87-0	(E)-2-Octenal	0.004	5.9	4.7	-	-	fatty green
1998/1/1	2-Furfural	9.562	<1	<1	-	-	sweet woody almond
104-61-0	2(3H)-Furanone, dihydro-5-pentyl-	0.0097	-	-	<1	23.4	coconut creamy sweet buttery
105-37-3	Ethyl propanoate	0.01	<1	<1	-	-	sweet fruity
106-32-1	Octanoic acid, ethyl ester	0.0193	-	-	<1	17.6	sweet fruity
119-36-8	Methyl salicylate	0.04	-	-	<1	<1	wintergreen mint
104-62-1	Formic acid, 2-phenylethyl ester	0.27	-	-	<1	<1	rose green hyacinth watercress herbal
141-78-6	Ethyl acetate	7.5	<1	<1	-	-	fruity sweet green
106-68-3	3-Octanone	0.0214	<1	<1	-	-	sweet mushroom
107-87-9	2-Pentanone	153	<1	<1	-	-	sweet fruity
110-43-0	2-Heptanone	0.14	<1	<1	-	-	fruity sweet
1629-58-9	1-Penten-3-one	0.0013	1.6	<1	-	-	pungent peppery onion
431-03-8	2,3-Butanedione	0.001	1.7	1.1	-	-	sweet creamy
4312-99-6	1-Octen-3-one(M)	0.000016	78.1	100	-	-	mushroom
67-64-1	Acetone	100	<1	<1	-	-	apple pear
78-93-3	2-Butanone	35.4002	<1	<1	-	-	fruity
111-27-3	1-Hexanol	0.2	<1	<1	<1	<1	fruity alcoholic sweet green
123-51-3	3-Methyl-1-butanol	0.22	<1	<1	-	-	alcoholic fruity
3391-86-4	1-Octen-3-ol	0.0015	11.4	9.9	<1	100	mushroom
515-00-4	Bicyclo[3.1.1]hept-2-ene-2-methanol, 6,6-dimethyl-	0.007	-	-	<1	<1	woody green
616-25-1	1-Penten-3-ol	0.3581	<1	<1	-	-	fruity green
626-93-7	2-Hexanol	6.7	-	-	<1	<1	fruity sweet green
64-17-5	Ethanol	2900	<1	<1	-	-	strong alcoholic
71-36-3	1-Butanol	0.5	<1	<1	-	-	sweet balsam whiskey
71-41-0	1-Pentanol	0.3581	<1	<1	-	-	sweet balsam
78-83-1	2-Methyl-1-propanol	8	<1	<1	-	-	ethereal winey cortex
3777-69-3	2-Pentylfuran	0.0058	7.6	<1	2.8	5.5	green beany vegetable

“-”, not detected.

## Data Availability

Not applicable.

## Supplementary Materials

The following supporting information can be downloaded at: https://www.mdpi.com/article/10.3390/foods11030272/s1, Figure S1: The HPLC-GPC spectrum of soybean beverage, Table S1: Free amino acid of the unfermented and fermented soy beverages with *N. aurantialba*, Table S2: Volatile compounds identified in samples by HS-GC-IMS, Table S3: Volatile compounds identified in samples by HS-SPME-GC-MS.

## References

[1] Sethi S., Tyagi S.K., Anurag R.K. (2016). Plant-based milk alternatives an emerging segment of functional beverages: A review. J. Food Sci. Technol..

[2] Aschemann-Witzel J., Gantriis R.F., Fraga P., Perez-Cueto F.J. (2020). Plant-based food and protein trend from a business perspective: Markets, consumers, and the challenges and opportunities in the future. Crit. Rev. Food Sci. Nutr..

[3] Freeland-Graves J.H., Nitzke S. (2013). Position of the academy of nutrition and dietetics: Total diet approach to healthy eating. J. Acad. Nutr. Diet..

[4] Paul A.A., Kumar S., Kumar V., Sharma R. (2020). Milk Analog: Plant based alternatives to conventional milk, production, potential and health concerns. Crit. Rev. Food Sci. Nutr..

[5] Yuan S., Chang S.K.-C. (2007). Selected odor compounds in soymilk as affected by chemical composition and lipoxygenases in five soybean materials. J. Agric. Food Chem..

[6] Wang B., Zhang Q., Zhang N., Bak K.H., Soladoye O.P., Aluko R.E., Fu Y., Zhang Y. (2021). Insights into formation, detection and removal of the beany flavor in soybean protein. Trends Food Sci. Technol..

[7] Wang J., Kuang H., Zhang Z., Yang Y., Yan L., Zhang M., Song S., Guan Y. (2020). Generation of seed lipoxygenase-free soybean using CRISPR-Cas9. Crop J..

[8] Ma L., Li B., Han F., Yan S., Wang L., Sun J. (2015). Evaluation of the chemical quality traits of soybean seeds, as related to sensory attributes of soymilk. Food Chem..

[9] Yu H., Liu R., Hu Y., Xu B. (2017). Flavor profiles of soymilk processed with four different processing technologies and 26 soybean cultivars grown in China. Int. J. Food Prop..

[10] Ghosh K., Ray M., Adak A., Halder S.K., Das A., Jana A., Parua S., Vágvölgyi C., Mohapatra P.K.D., Pati B.R. (2015). Role of probiotic Lactobacillus fermentum KKL1 in the preparation of a rice based fermented beverage. Bioresour. Technol..

[11] Nedele A.-K., Gross S., Rigling M., Zhang Y. (2020). Reduction of green off-flavor compounds: Comparison of key odorants during fermentation of soy drink with Lycoperdon pyriforme. Food Chem..

[12] Mäkinen O.E., Wanhalinna V., Zannini E., Arendt E.K. (2016). Foods for special dietary needs: Non-dairy plant-based milk substitutes and fermented dairy-type products. Crit. Rev. Food Sci. Nutr..

[13] Cao Z.-H., Green-Johnson J.M., Buckley N.D., Lin Q.-Y. (2019). Bioactivity of soy-based fermented foods: A review. Biotechnol. Adv..

[14] Zhu Y.-Y., Thakur K., Feng J.-Y., Cai J.-S., Zhang J.-G., Hu F., Wei Z.-J. (2020). B-vitamin enriched fermented soymilk: A novel strategy for soy-based functional foods development. Trends Food Sci. Technol..

[15] Shon Y.-H., Nam K.-S. (2004). Inhibition of cytochrome P450 isozymes and ornithine decarboxylase activities by polysaccharides from soybeans fermented with Phellinus igniarius or Agrocybe cylindracea. Biotechnol. Lett..

[16] Yang H., Zhang L. (2009). Changes in some components of soymilk during fermentation with the basidiomycete Ganoderma lucidum. Food Chem..

[17] Du X., Zhang Y., Mu H., Lv Z., Yang Y., Zhang J. (2015). Structural elucidation and antioxidant activity of a novel polysaccharide (TAPB1) from Tremella aurantialba. Food Hydrocoll..

[18] Yuan Q., Zhang X., Ma M., Long T., Xiao C., Zhang J., Liu J., Zhao L. (2020). Immunoenhancing glucuronoxylomannan from Tremella aurantialba Bandoni et Zang and its low-molecular-weight fractions by radical depolymerization: Properties, structures and effects on macrophages. Carbohydr. Polym..

[19] Deng C., Sun Y., Fu H., Zhang S., Chen J., Xu X. (2016). Antioxidant and immunostimulatory activities of polysaccharides extracted from Tremella aurantialba mycelia. Mol. Med. Rep..

[20] Zhang Z., Lian B., Huang D., Cui F. (2009). Compare activities on regulating lipid-metabolism and reducing oxidative stress of diabetic rats of Tremella aurantialba broth’s extract (TBE) with its mycelia polysaccharides (TMP). J. Food Sci..

[21] Guo Y.-J., Deng G.-F., Xu X.-R., Wu S., Li S., Xia E.-Q., Li F., Chen F., Ling W.-H., Li H.-B. (2012). Antioxidant capacities, phenolic compounds and polysaccharide contents of 49 edible macro-fungi. Food Funct..

[22] Bandoni R.J. (1963). Conjugation in Tremella mesenterica. Can. J. Bot..

[23] Ingold C. (1982). Basidiospore germination in Tremella foliacea. Trans. Br. Mycol. Soc..

[24] Sun T., Wang R., Sun D., Li S., Xu H., Qiu Y., Lei P., Sun L., Xu X., Zhu Y. (2020). High-efficiency production of Tremella aurantialba polysaccharide through basidiospore fermentation. Bioresour. Technol..

[25] Sun T., Zhang Y., Jiang H., Yang K., Wang S., Wang R., Li S., Lei P., Xu H., Qiu Y. (2022). Whole genome sequencing and annotation of naematelia aurantialba (Basidiomycota, Edible-Medicinal Fungi). J. Fungi.

[26] Dai C., Huang X., Lv R., Zhang Z., Sun J., Aheto J.H. (2018). Analysis of volatile compounds of Tremella aurantialba fermentation via electronic nose and HS-SPME-GC-MS. J. Food Saf..

[27] Hassan K.A., Mujtaba M.A. (2019). Antibacterial efficacy of garlic oil nano-emulsion. AIMS Agric. Food.

[28] Li H., Xu H., Li S., Feng X., Xu H., Ouyang P. (2011). Effects of dissolved oxygen and shear stress on the synthesis and molecular weight of welan gum produced from *Alcaligenes* sp. CGMCC2428. Process Biochem..

[29] Oliveira L.C., Schmiele M., Steel C.J. (2017). Development of whole grain wheat flour extruded cereal and process impacts on color, expansion, and dry and bowl-life texture. LWT.

[30] Jiang K., Tang B., Wang Q., Xu Z., Sun L., Ma J., Li S., Xu H., Lei P. (2019). The bio-processing of soybean dregs by solid state fermentation using a poly γ-glutamic acid producing strain and its effect as feed additive. Bioresour. Technol..

[31] Yang H., Zhang L., Xiao G., Feng J., Zhou H., Huang F. (2015). Changes in some nutritional components of soymilk during fermentation by the culinary and medicinal mushroom Grifola frondosa. LWT.

[32] Jaganath I.B., Crozier A. (2010). Dietary flavonoids and phenolic compounds. Plant Phenolics Hum. Health Biochem. Nutr. Pharmacol..

[33] Gu J., Zhang H., Zhang J., Wen C., Ma H., Duan Y., He Y. (2020). Preparation, characterization and bioactivity of polysaccharide fractions from *Sagittaria sagittifolia* L.. Carbohydr. Polym..

[34] Su Y., Li L. (2020). Structural characterization and antioxidant activity of polysaccharide from four auriculariales. Carbohydr. Polym..

[35] Phuhongsung P., Zhang M., Bhandari B. (2020). 4D printing of products based on soy protein isolate via microwave heating for flavor development. Food Res. Int..

[36] Menis-Henrique M.E.C. (2020). Methodologies to advance the understanding of flavor chemistry. Curr. Opin. Food Sci..

[37] Li M., Du H., Lin S. (2021). Flavor changes of tricholoma matsutake singer under different processing conditions by using HS-GC-IMS. Foods.

[38] Chen Y., Li P., Liao L., Qin Y., Jiang L., Liu Y. (2021). Characteristic fingerprints and volatile flavor compound variations in Liuyang Douchi during fermentation via HS-GC-IMS and HS-SPME-GC-MS. Food Chem..

[39] Yan Y., Wang M., Jin B., Yang J., Li S. (2021). Performance evaluation and microbial community analysis of the biofilter for removing grease and volatile organic compounds in the kitchen exhaust fume. Bioresour. Technol..

[40] Tieman D., Zhu G., Resende M.F., Lin T., Nguyen C., Bies D., Rambla J.L., Beltran K.S.O., Taylor M., Zhang B. (2017). A chemical genetic roadmap to improved tomato flavor. Science.

[41] Yi C., Li Y., Zhu H., Liu Y., Quan K. (2021). Effect of Lactobacillus plantarum fermentation on the volatile flavors of mung beans. LWT.

[42] Hothorn T., Zeileis A. (2015). Partykit: A modular toolkit for recursive partytioning in R. J. Mach. Learn. Res..

[43] Shi F., Tian X., McClements D.J., Chang Y., Shen J., Xue C. (2021). Influence of molecular weight of an anionic marine polysaccharide (sulfated fucan) on the stability and digestibility of multilayer emulsions: Establishment of structure-function relationships. Food Hydrocoll..

[44] Wei Y., Cai Z., Wu M., Guo Y., Tao R., Li R., Wang P., Ma A., Zhang H. (2020). Comparative studies on the stabilization of pea protein dispersions by using various polysaccharides. Food Hydrocoll..

[45] Sun W., Zheng Y., Chen S., Chen J., Zhang H., Fang H., Ye X., Tian J. (2021). Applications of polysaccharides as stabilizers in acidified milks. Food Rev. Int..

[46] Shao P., Feng J., Sun P., Xiang N., Lu B., Qiu D. (2020). Recent advances in improving stability of food emulsion by plant polysaccharides. Food Res. Int..

[47] Martínez-Monteagudo S.I., Kamat S., Patel N., Konuklar G., Rangavajla N., Balasubramaniam V. (2017). Improvements in emulsion stability of dairy beverages treated by high pressure homogenization: A pilot-scale feasibility study. J. Food Eng..

[48] Adediran A., Aforijiku S. (2020). Carbohydrate fermentation profile and physiological studies of lactic acid bacteria from native raw cow milk. J. Adv. Microbiol. Res..

[49] Niyigaba T., Liu D., Habimana J.D.D. (2021). The extraction, functionalities and applications of plant polysaccharides in fermented foods: A review. Foods.

[50] Lin C., Lin Y., Xiao J., Lan Y., Cao Y., Chen Y. (2021). Effect of momordica saponin-and cyclocarya paliurus polysaccharide-enriched beverages on oxidative stress and fat accumulation in caenorhabditis elegans. J. Sci. Food Agric..

[51] Lee M.-H., Kim M., Kim M., Kwak J.H., Chang D.H., Yu W.K., Lee S.-H., Lee J.H. (2016). Consumption of dairy yogurt with the polysaccharide rhamnogalacturonan from the peel of the Korean citrus hallabong enhances immune function and attenuates the inflammatory response. Food Funct..

[52] Zhao J., Liu W., Chen D., Zhou C., Song Y., Zhang Y., Ni Y., Li Q. (2015). Microbiological and physicochemical analysis of pumpkin juice fermentation by the basidiomycetous fungus Ganoderma lucidum. J. Food Sci..

[53] Islam T., Yu X., Xu B. (2016). Phenolic profiles, antioxidant capacities and metal chelating ability of edible mushrooms commonly consumed in China. LWT.

[54] Puchau B., Zulet M.A., de Echávarri A.G., Hermsdorff H.H.M., Martínez J.A. (2009). Dietary total antioxidant capacity: A novel indicator of diet quality in healthy young adults. J. Am. Coll. Nutr..

[55] Du X., Zhang J., Lv Z., Ye L., Yang Y., Tang Q. (2014). Chemical modification of an acidic polysaccharide (TAPA1) from Tremella aurantialba and potential biological activities. Food Chem..

[56] Rodríguez-Maecker R., Vyhmeister E., Meisen S., Martinez A.R., Kuklya A., Telgheder U. (2017). Identification of terpenes and essential oils by means of static headspace gas chromatography-ion mobility spectrometry. Anal. Bioanal. Chem..

[57] Chen Y. (2015). Effects of Micronization, Ethanol Washing, and Enzymatic Hydrolysis Processing Alone or in Combination on Trypsin Inhibitors, Lipoxygenase Activities and Selected “Beany” Flavour Related Compounds in Soybean Flour. Master’s Thesis.

[58] Vermeulen N., Czerny M., Gänzle M.G., Schieberle P., Vogel R.F. (2007). Reduction of (E)-2-nonenal and (E, E)-2, 4-decadienal during sourdough fermentation. J. Cereal Sci..

[59] El Youssef C., Bonnarme P., Fraud S., Péron A.-C., Helinck S., Landaud S. (2020). Sensory improvement of a pea protein-based product using microbial co-cultures of lactic acid bacteria and yeasts. Foods.

[60] Brunton N.P., Cronin D.A., Monahan F.J., Durcan R. (2000). A comparison of solid-phase microextraction (SPME) fibres for measurement of hexanal and pentanal in cooked turkey. Food Chem..

[61] Guo Y., Chen D., Dong Y., Ju H., Wu C., Lin S. (2018). Characteristic volatiles fingerprints and changes of volatile compounds in fresh and dried Tricholoma matsutake Singer by HS-GC-IMS and HS-SPME-GC–MS. J. Chromatogr. B.

[62] Ames J.M. (1990). Control of the Maillard reaction in food systems. Trends Food Sci. Technol..

[63] Hiraide M., Miyazaki Y., Shibata Y. (2004). The smell and odorous components of dried shiitake mushroom, Lentinula edodes I: Relationship between sensory evaluations and amounts of odorous components. J. Wood Sci..

[64] Tian Y., Zhao Y., Huang J., Zeng H., Zheng B. (2016). Effects of different drying methods on the product quality and volatile compounds of whole shiitake mushrooms. Food Chem..

[65] Hidalgo F.J., Zamora R. (2019). Formation of phenylacetic acid and benzaldehyde by degradation of phenylalanine in the presence of lipid hydroperoxides: New routes in the amino acid degradation pathways initiated by lipid oxidation products. Food Chem. X.

[66] Mennella J.A., Bobowski N.K., Reed D.R. (2016). The development of sweet taste: From biology to hedonics. Rev. Endocr. Metab. Disord..

[67] Wang Y., Tao Y., Zhang X., Shao S., Han Y., Chu D.-T., Xie G., Ye X. (2019). Metabolic profile of ginkgo kernel juice fermented with lactic aicd bacteria: A potential way to degrade ginkgolic acids and enrich terpene lactones and phenolics. Process Biochem..

[68] De Filippis F., Troise A.D., Vitaglione P., Ercolini D. (2018). Different temperatures select distinctive acetic acid bacteria species and promotes organic acids production during Kombucha tea fermentation. Food Microbiol..

[69] Berenguer M., Vegara S., Barrajón E., Saura D., Valero M., Martí N. (2016). Physicochemical characterization of pomegranate wines fermented with three different Saccharomyces cerevisiae yeast strains. Food Chem..

[70] Cai W., Tang F., Zhao X., Guo Z., Zhang Z., Dong Y., Shan C. (2019). Different lactic acid bacteria strains affecting the flavor profile of fermented jujube juice. J. Food Process. Preserv..

