# Supplementary material for: Nutritional Function and Flavor Evaluation of a New Soybean Beverage Based on Naematelia aurantialba Fermentation
Source: Foods. 2022 Jan 20;11(3):272. doi: 10.3390/foods11030272 (PMC8834624; doi:10.3390/foods11030272)
Supplement: Supplementary file 1 [file foods-11-00272-s001.zip › foods-1544806-supplementary.pdf]

Supporting Information

Nutritional function evaluation and flavor of a new soybean beverage based on *Tremella aurantialba* fermentation

Tao Sun[1], Hao Jiang[1], Kai Yang[1], Xingkai Li[1], Shiyu Wang[1], Haoyu Yao[1], Rui Wang[1], Sha Li[1], Yian Gu[1], Peng Lei[1, *], Hong Xu[1, *] and Dafeng Sun[2].

1. State Key Laboratory of Materials-Oriented Chemical Engineering, College of Food Science and Light Industry, Nanjing Tech University, Nanjing 211816, China.; sun-tao@njtech.edu.cn (T.S.); jianghao@njtech.edu.cn (H.J.); 201961119005@njtech.edu.cn (K.Y.); 201921019081@njtech.edu.cn (S. W.); 201921019097@njtech.edu.cn(H.Y.); ruiwang2013@njtech.edu.cn (R.W.); lisha@njtech.edu.cn (S.L.); yian.gu@hotmail.com(Y.G.) lei-peng@njtech.edu.cn (P.L.); xuh@njtech.edu.cn (H.X.).

2. Kunming Edible Fungi Institute of All China Federation of Supply and Marketing Cooperatives, Kunming 650032, Yunnan, China; sdafeng@163.com (D.S.).

* Correspondence: lei-peng@njtech.edu.cn; Tel.: +86 18761681790, xuh@njtech.edu.cn; Tel: +86-25-58139433.

Table S1. Free amino acid of the unfermented and fermented soy beverages with *N. aurantialba*.

	UFSB free amino acid content (μg/mL)	FSB free amino acid content (μg/mL)
	non-essential amino acid	
Asp	6.83±0.02	3.70±0.02
Glu	1.90±0.01	3.41±0.02
Ser	3.90±0.03	1.99±0.05
His	10.05±0.06	8.47±0.08
Gly	1.25±0.02	1.00±0.04
Arg	2.37±0.03	2.87±0.03
Ala	1.33±0.04	2.06±0.04
Tyr	0.26±0.03	0.67±0.03
Cys	0.00	1.70±0.07
Pro	0.00	0.88±0.02
	essential amino acid	
Val	0.75±0.02	1.63±0.02
Met	0.34±0.04	0.24±0.01
Phe	0.15±0.03	0.46±0.05
Ile	0.48±0.05	0.72±0.02
Leu	0.55±0.08	0.78±0.01
Lys	0.51±0.09	0.79±0.03
Thr	1.35±0.02	1.47±0.05
Total	32.02±0.11	32.84±0.08

Table S2. Volatile compounds identified in samples by HS-GC-IMS.

Count	Compounds	RI	Rt [sec]	Dt [a.u.]	Comment	Peak intensity		Relative amount (%)		Odor description
						UFSB	FSB	UFSB	FSB	
					Aldehydes					
1	2,4-Decadienal	1285	1352	1.42		740±21.91^{a}	1201±37.16^{b}	1.20±0.011^{A}	1.03±0.054^{B}	fatty green
2	(E)-2-Decenal	1233	1148	1.48	Monomer	317±26.54^{a}	615±29.05^{b}	0.520±0.052^{A}	0.530±0.025^{A}	fatty, fish, hay
3	(E)-2-Nonenal	1147	879	1.41	Dimer	173±20.88^{a}	389±31.91^{b}	0.280±0.052^{A}	0.330±0.030^{A}	fatty green
4	n-Nonanal	1106	772	1.48	Monomer	1436±74.18^{a}	2057±83.4^{b}	2.33±0.080^{A}	1.76±0.057^{B}	rose fresh fruity
5	n-Nonanal	1106	772	1.94	Dimer	193±22.51^{a}	321±19.22^{b}	0.310±0.035^{A}	0.280±0.016^{A}	
6	(E)-2-Octenal	1072	695	1.34	Monomer	1761±256.92^{a}	5182±296.20^{b}	2.85±0.430^{A}	4.44±0.239^{B}	fatty green
7	(E)-2-Octenal	1072	694	1.81	Dimer	297±5.22^{a}	2253±226.64^{b}	0.480±0.023^{A}	1.93±0.205^{B}	
8	(E, E)-2,4-heptadienal	1036	621	1.19	Monomer	385±25.93^{a}	1234±106.94^{b}	0.620±0.036^{A}	1.06±0.095^{B}	fatty green
9	(E, E)-2,4-heptadienal	1035	619	1.61	Dimer	56.3±2.3^{a}	437±36.09^{b}	0.090±0.002^{A}	0.370±0.032^{B}	
10	2,4-Heptadienal	1019	590	1.20		204±49.78^{a}	450±122.55^{a}	0.330±0.092^{A}	0.380±0.124^{A}	green fruity
11	Octanal	1015	581	1.42	Monomer	742±41.18^{a}	2059±13.37^{b}	1.20±0.044^{A}	1.77±0.029^{B}	green fatty
12	Octanal	1014	580	1.82	Dimer	128±11.04^{a}	762±39.43^{b}	0.210±0.021^{A}	0.650±0.037^{B}	
18	(E)-2-Heptenal	964	485	1.26	Monomer	2217±177.63^{a}	5097±26.60^{b}	3.59±0.245^{A}	4.37±0.068^{B}	green fatty
19	(E)-2-Heptenal	964	484	1.66	Dimer	1440±60.33^{a}	11330±155.74^{b}	2.34±0.088^{A}	9.72±0.031^{B}	

20	Benzaldehyde	981	518	1.15		556±89.53[a]	762±130.70[a]	0.900±0.159[A]	0.650±0.128[A]	bitter almond, sweet cherry
21	Heptanal	905	387	1.36	Monomer	897±80.25[a]	1549±7.10[b]	1.45±0.118[A]	1.33±0.020[B]	fatty green
22	Heptanal	904	385	1.69	Dimer	360±19.69[a]	1483±32.05[b]	0.580±0.023[A]	1.27±0.035[B]	
27	(E)-2-hexenal	858	325	1.18	Monomer	2191±75.17[a]	1853±15.51[b]	3.55±0.057[A]	1.59±0.037[B]	green fatty
28	(E)-2-hexenal	855	322	1.51	Dimer	2210±161.95[a]	3358±34.75[b]	3.58±0.217[A]	2.88±0.062[B]	
29	2-Furfural	833	298	1.09	Monomer	158±16.52[a]	301±98.10[a]	0.260±0.028[A]	0.260±0.100[A]	sweet woody almond
30	2-Furfural	831	295	1.33	Dimer	24.2±0.75[a]	152±35.62[b]	0.040±0.0003[A]	0.130±0.035[B]	
31	hexanal	796	260	1.55		10645±125.39[a]	8606±154.3[b]	17.3±0.708[A]	7.38±0.039[B]	green fatty
32	(E)-2-pentenal	756	224	1.36		783±62.21[a]	1223±16.05[b]	1.27±0.095[A]	1.05±0.002[B]	green fruity
33	pentanal	699	180	1.19	Monomer	919±15.54[a]	721±24.50[b]	1.49±0.074[A]	0.620±0.030[B]	fruity berry
34	pentanal	701	182	1.43	Dimer	449±20.92[a]	1976±79.70[b]	0.730±0.02[A]	1.70±0.109[B]	
38	butanal	605	135	1.29		546±29.24[a]	1047±12.40[b]	0.890±0.039[A]	0.900±0.021[A]	pungent cocoa green
54	benzeneacetaldehyde	1071	693	1.26		140±5.35[a]	303±16.20[b]	0.230±0.004[A]	0.260±0.018[A]	green sweet cocoa
						Alcohols				

13	1-Octen-3-ol	998	552	1.16		1268±68.22^{a}	4053±20.6^{b}	2.06±0.072^{A}	3.48±0.082^{B}	mushroom
25	1-hexanol	886	359	1.32	Monomer	1574±47.17^{a}	585±52.98^{b}	2.55±0.032^{A}	0.500±0.065^{B}	fruity alcoholic sweet green
26	1-hexanol	884	357	1.64	Dimer	367±38.96^{a}	96.0±13.85^{b}	0.600±0.094^{A}	0.080±0.016^{B}	
35	2-Methyl-1-propanol	632	146	1.37		153±9.48^{a}	6402±25.83^{b}	0.250±0.012^{A}	5.49±0.124^{B}	ethereal winey
40	ethanol	483	94.5	1.05	Monomer	6453±234.77^{a}	6418±58.28^{a}	10.5±0.367^{A}	5.51±0.062^{B}	cortex strong alcoholic
41	ethanol	486	95.2	1.14	Dimer	1222±110.34^{a}	2024±47.70^{b}	1.99±0.251^{A}	1.74±0.080^{A}	
50	1-butanol	668	163	1.17	Monomer	264±15.18^{a}	1284±4.06^{b}	0.430±0.042^{A}	1.10±0.023^{B}	sweet balsam whiskey
51	1-butanol	667	162	1.39	Dimer	21.9±1.24^{a}	1786±14.72^{b}	0.040±0.004^{A}	1.53±0.017^{B}	
52	3-Methyl-1-butanol	744	214	1.24	Monomer	834±50.95^{a}	1151±12.42^{b}	1.35±0.059^{A}	0.990±0.030^{B}	alcoholic fruity
53	3-Methyl-1-butanol	745	215	1.50	Dimer	254±9.75^{a}	8971±70.43^{b}	0.410±0.023^{A}	7.70±0.062^{B}	
43	1-penten-3-ol	692	175	1.34		276±10.76^{a}	1046±11.24^{b}	0.450±0.007^{A}	0.900±0.004^{B}	fruity green
44	1-Pentanol	777	243	1.51		403±24.21^{a}	775±16.71^{b}	0.650±0.044^{A}	0.660±0.011^{A}	sweet balsam
						Furan				
14	2-Pentylfuran	999	553	1.25		3254±70.78^{a}	1267±143.74^{b}	5.28±0.040^{A}	1.09±0.132^{B}	green beany vegetable

					Ketones					
15	3-Octanone	993	543	1.31		104±12.77[a]	236±4.21[b]	0.170±0.020[A]	0.200±0.002[A]	sweet mushroom
16	1-Octen-3-one	987	531	1.27	Monomer	92.6±4.09[a]	436±32.05[b]	0.150±0.007[A]	0.370±0.040[B]	mushroom
17	1-Octen-3-one	986	528	1.68	Dimer	71.1±35.86[a]	687±38.39[b]	0.120±0.076[A]	0.590±0.050[B]	
23	2-heptanone	895	372	1.26	Monomer	460±17.91[a]	1056±18.17[b]	0.750±0.017[A]	0.910±0.008[B]	fruity sweet
24	2-heptanone	896	373	1.63	Dimer	707±70.41[a]	2087±51.44[b]	1.14±0.106[A]	1.79±0.037[B]	
39	2-Butanone	592	130	1.24		123±22.76[a]	384±10.14[b]	0.200±0.042[A]	0.330±0.014[B]	fruity
42	Acetone	512	103	1.13		4769±370.05[a]	5842±76.10[b]	7.75±0.893[A]	5.01±0.168[B]	apple pear
43	1-penten-3-one	689	173	1.08		151±15.67[a]	118±1.43[b]	0.250±0.039[A]	0.100±0.003[B]	pungent peppery onion
47	2,3-butanedione	591	130	1.17		127±26.12[a]	309±3.37[b]	0.210±0.049[A]	0.270±0.008[A]	sweet creamy
48	2-pentanone	687	172	1.12	Monomer	49.6±2.71[a]	129±2.82[b]	0.080±0.006[A]	0.110±0.001[B]	sweet fruity
49	2-pentanone	691	175	1.36	Dimer	299±8.15[a]	953±23.82[b]	0.480±0.002[A]	0.820±0.011[B]	
					Esters					
36	ethyl acetate	606	136	1.10	Monomer	1969±29.47[a]	902±8.44[b]	3.20±0.154[A]	0.770±0.022[B]	fruity sweet green
37	ethyl acetate	612	138	1.33	Dimer	3450±171.87[a]	3524±43.80[a]	5.60±0.213[A]	3.02±0.054[B]	
45	ethyl propanoate	710	188	1.15		73.5±8.31[a]	63.0±20.47[a]	0.120±0.014[A]	0.050±0.021[B]	sweet fruity

Rt: Represents the retention time in the capillary GC column; RI: Represents the retention index calculated using n-ketones C4–C9 as external standard on FS-SE-54-CB-1 column; Dt: Represents the drift time in the drift tube; Each value is expressed as mean ± SD (n = 3); For Peak intensity: Means with different lowercase letters within a row indicate significant differences ($P < 0.05$); For Relative amount: Means with different capital letters within a row indicate significant differences ($P < 0.05$); Relative amount, the percentage of each compound peak intensity to total peak intensity of all compounds.

Table S3. Volatile compounds identified in samples by HS-SPME-GC-MS.

No.	RT	Compounds	RI	NIST_RI	Odor description	Peak intensity		Relative amount (%)	
						UFSB	FSB	UFSB	FSB
Heterocyclic compound									
1	3.06	Oxetane, 3-(1-methylethyl)-	718.12	664	-	ND	1621445±196936	ND	2.53±0.418
5	5.56	2,4-Imidazolidinedione, 1-methyl-	858.25	1047	-	539509±23340[a]	534025±21412[a]	0.96±0.054[A]	0.83±0.076[A]
9	8.91	4H-Pyran-4-one	986.06	972	-	768681±104164[a]	798350±23684[a]	1.37±0.089[A]	1.24±0.119[A]
10	8.96	2-pentyl-furan	987.89	993	green earthy beany	6498125±534065[a]	712224±128211[b]	11.58±0.293[A]	1.12±0.265[B]
14	10.8		1051.11	1048	roasted	186144±31660[a]	125155±7559[b]	0.33±0.027[A]	0.20±0.024[B]
21	12.25	Thiophene, 2-butyl-	1100.42	1093	fruity floral fried chicken	784151±1015460[a]	122485±20612[b]	1.47±1.949[A]	0.19±0.043[B]
22	13.5	Thiophene, 2-pentyl-	1143.43	1090	fruit, sweet	ND	628882±20192	ND	0.98±0.075
26	14.78	Thiophene, 2-hexyl-	1187.4	1277	floral fruity gassy	ND	251010±32886	ND	0.39±0.03
30	15.21	4-Formyl-3,5-dimethyl-1H-pyrrole-2-carbonitrile	1202.07	1501	-	66652±13900[a]	49432±9272[a]	0.12±0.020[A]	0.08±0.019[A]

36	17.91	1-(2-furanyl)- 1-Pentanone	1299.53	1176	sweet caramel	184692±27820[a]	557517±87545[b]	0.33±0.015[A]	0.86±0.073[B]
37	18.2	3,5-Dimethyl-1-butylpyrazole	1310.26	1192	-	215228±10809[a]	187677±29806[a]	0.39±0.053[A]	0.29±0.053[B]
					Alcohol				
2	3.12	1-Butanol, 2-methyl-, (S)-	722.04	739	ethereal fresh	ND	2005383±259507	ND	3.12±0.44
3	3.43	2-Hexanol	743.84	801	fruity sweet green	32880±3843[a]	47649±10827[b]	0.06±0.006[A]	0.07±0.012[A]
6	5.67	1-Hexanol	863.1	851	fruity alcoholic sweet green	6028069±441951[a]	6629745±1238254[a]	10.76±0.558[A]	10.23±1.162[A]
8	8.67	1-Octen-3-ol	977.65	982	mushroom	984538±121842[a]	3477799±1627475[b]	1.76±0.21[A]	5.3±0.713[B]
					Ketone				
4	4.67	Norbornadieone	819.37	913	-	454614±76709[a]	944981±145802[b]	0.81±0.08[A]	1.47±0.227[B]
20	12	3,5-Octadien-2-one	1091.64	1095	fruity fatty	279255±39036[a]	268846±23341[a]	0.50±0.025[A]	0.42±0.017[B]
35	16.82	3(2H)-Furanone, 2-hexyl-5-methyl-	1260.01	1446	-	404040±35361[a]	477153±13919[b]	0.72±0.026[A]	0.74±0.032[B]
43	21.44	Pyrolo[3,2-d]pyrimidin-2,4(1H,3H)-dione	1435.18	1475	-	85937±12521[a]	60824±5944[b]	0.15±0.01[A]	0.1±0.015[B]
					Aldehyde				
7	8.11	Benzaldehyde	957.34	960	bitter almond , sweet cherry	1346850±149896[a]	16021725±803440[b]	2.4±0.19[A]	24.88±0.713[B]

11	9.58	(E, E)-2,4-heptadienal	1009.64	1011	fatty green	458123±83423^{a}	292071±65426^{b}	0.82±0.162^{A}	0.45±0.074^{B}
15	10.95	2-octenal	1056.04	1060	fatty green herbal	510065±53147^{a}	150411±12114^{b}	0.91±0.006^{A}	0.23±0.006^{B}
24	14.01	4-ethyl-benzaldehyde	1160.8	1171	bitter almond	316945±43024^{a}	72065±1910^{b}	0.56±0.018^{A}	0.11±0.01^{B}
33	15.48	Benzaldehyde, 2,5-dimethyl-	1212.05	1208	-	3877716±724892^{a}	1918279±172111^{b}	6.87±0.59^{A}	3±0.443^{B}
39	18.34	2,4-Decadienal, (E,E)-	1315.79	1317	fatty green	1186788±64214^{a}	57711±9133^{b}	2.12±0.098^{A}	0.09±0.02^{B}
					Aromatics				
12	9.64	Benzene, 1,4-dichloro-	1011.56	1021	-	32707±5039^{a}	36108±850^{a}	0.06±0.003^{A}	0.06±0.005^{A}
16	11.02	Benzene, (1-methylpropyl)-	1058.49	994	-	ND	36298±3723	ND	0.06±0.009
19	11.88	Benzene, 1,2,3,5-tetramethyl-	1087.72	1133	-	147736±17987^{a}	188370±24204^{b}	0.26±0.008^{A}	0.29±0.041^{B}
23	13.53	Indan, 1-methyl-	1144.47	1073	bitter almond sweet anise	323150±108105^{a}	104445±10183^{b}	0.57±0.157^{A}	0.16±0.026^{B}
25	14.6	Naphthalene	1181.25	1214	tar, camphor	928181±100887	798786±58287	1.65±0.029^{A}	1.25±0.151^{B}
					Olefin				
13	9.94	1,3-Cyclopentadiene, 1,2,3,4,5-pentamethyl-	1021.8	983	-	790158±157387^{a}	1225832±123920^{b}	1.4±0.184^{A}	1.92±0.31^{A}

16	11.52	1,3-Cyclohexadiene, 5-butyl-	1075.34	1043	-	141681±4887[a]	33817±4579[b]	0.25±0.028[A]	0.05±0.01[B]
34	15.77	1-Hexen, 2-(p-anisyl)-5-methyl-	1222.49	1482	-	241493±25517[a]	135919±18465[b]	0.43±0.02[A]	0.21±0.041[B]
Ester									
17	11.36	Formic acid, octyl ester	1069.94	1114	fruity rose orange	1323369±76621[a]	1754644±708363[a]	2.37±0.259[A]	2.68±0.877[A]
27	14.85	Methyl salicylate	1189.63	1234	wintergreen mint	134718±47046[a]	80900±8110[b]	0.24±0.096[A]	0.13±0.009[B]
28	15	Octanoic acid, ethyl ester	1194.8	1198	sweet fruity	4720782±406796[a]	7761488±1037991[b]	8.41±0.32[A]	12.01±0.754[B]
32	15.48	Formic acid, 2-phenylethyl ester	1211.84	1178	rose green hyacinth watercress herbal	484348±92725[a]	229852±17038[b]	0.86±0.074[A]	0.36±0.046[B]
40	19.39	2(3H)-Furanone, dihydro-5-pentyl-	1355.43	1366	coconut creamy sweet buttery	120699±23131[a]	5154713±83079[b]	0.21±0.031[A]	8.03±0.606[B]
41	19.75	n-Caproic acid vinyl ester	1369.16	1244	-	335122±17289[a]	413055±38253[b]	0.6±0.033[A]	0.64±0.031[B]
					Alkanes				
29	15.13	Dodecane	1199.28	1200	Gasoline alkane	7905±2025[a]	9031±992[a]	0.01±0.003[A]	0.01±0.002[A]
42	20.55	Tetradecane	1399.48	1400	gasoline Waxy	465049±66561[a]	436303±79678[a]	0.83±0.114[A]	0.68±0.165[A]

44	22.14	Tetradecane, 2-methyl-	1463.14	1463	-	56087±8907[a]	59921±14154[a]	0.1±0.017[A]	0.09±0.027[A]
45	22.73	Hexadecane, 2,6,11,15-tetramethyl-	1486.84	1753	-	394580±8907[a]	389904±24894[a]	0.7±0.065[A]	0.61±0.074[A]
46	23.04	Pentadecane	1499.39	1500	Waxy	6863180±1464858[a]	5330964±312569[a]	12.14±1.521[A]	8.3±0.831[B]
47	23.71	5,5-Diethyltridecane	1527.74	1627	-	27610±5280[a]	23082±2353[a]	0.05±0.005[A]	0.04±0.006[A]
48	24.55	Pentadecane, 2-methyl-	1563.07	1548	-	176855±30208[a]	135831±25561[a]	0.31±0.032[A]	0.21±0.052[B]
49	25.4	Hexadecane	1599.19	1600	-	434540±67488[a]	338999±64296[a]	0.77±0.063[A]	0.53±0.126[B]
50	26.98	Heptadecane, 2-methyl-（2-methylheptadecane）	1669.7	1765	Lemon balm	19613±3430[a]	45842±10061[b]	0.03±0.003[A]	0.07±0.02[B]
51	27.53	Heneicosane	1693.92	2100	waxy	98962±17257[a]	123312±14607[a]	0.18±0.02[A]	0.19±0.034[A]
52	27.6	Eicosane	1697.36	2000	waxy	632134±98142[a]	674051±127289[a]	1.12±0.106[A]	1.06±0.25[A]
53	27.72	7-Methyl-octadecane	1702.76	1842	-	384176±53939[a]	442141±61854[a]	0.68±0.086[A]	0.69±0.137[A]
54	29.78	Octadecane	1799.22	1800	-	186318±26119[a]	214514±82728[a]	0.33±0.018[A]	0.34±0.143[A]
					Terpenes				

31	15.31	Bicyclo[3.1.1]hept-2-ene-2-methanol, 6,6-dimethyl-	1205.69	1195	woody green	13429±410[a]	28839±4404b[a]	0.02±0.003[A]	0.05±0.009[B]
					Phenol				
38	18.26	Phenol, 2-(1-methylpropyl)-	1312.66	1248	-	11488361±1373803[a]	232656±8952[b]	20.42±0.36[A]	0.36±0.02[B]

Rt: Represents the retention time in the capillary GC column; RI: Retention Index using a DB-5MS capillary column; Each value is expressed as mean ± SD (n = 3); For Peak intensity: Means with different lowercase letters within a row indicate significant differences ($P < 0.05$); For Relative amount: Means with different capital letters within a row indicate significant differences ($P < 0.05$); Relative amount, the percentage of each compound peak intensity to total peak intensity of all compounds; ND, not detected; "-", not described.

Figure S1

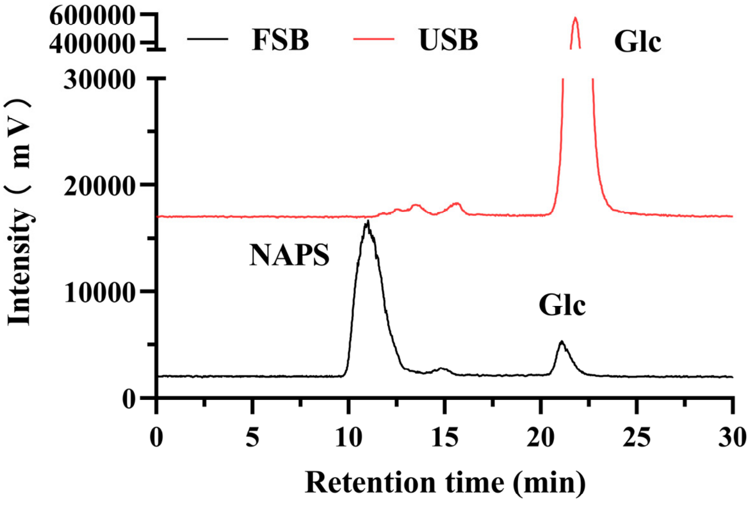

Figure S1. HPLC-GPC spectrum of soybean beverage.
